# Multi-Agent LLMs for Occupational Profiling: Psychometric Validation on 1636 Chinese Occupations

**DOI:** 10.3390/bs16071064

**Published:** 2026-06-26

**Authors:** Yuting Han, Xiaoyang Luo, Feng Ji, Xiang Kong

**Affiliations:** 1 Cognitive Science and Allied Health School, Institute of Life and Health Sciences, Key Laboratory of Language and Cognitive Science (Ministry of Education), Beijing Language and Culture University, Beijing 100083, China; hanyuting716@gmail.com; 2 School of Information Science, Speech Acquisition and Intelligent Technology Laboratory (SAIT), Beijing Language and Culture University, Beijing 100083, China; 202211590399@stu.blcu.edu.cn; 3 Department of Applied Psychology and Human Development, University of Toronto, Toronto, ON M5S 1V6, Canada; f.ji@utoronto.ca; 4 School of Linguistics and Language Resources, Beijing Language and Culture University, Beijing 100083, China

**Keywords:** multi-agent LLM, LLM-as-rater, psychometric validation, occupational profiling, inter-rater reliability

## Abstract

Occupation-level psychological profiles, such as RIASEC interests and Big Five personality, underpin career counseling, person–job matching, and workforce research, but building them at scale has been expensive and limited to a few national taxonomies. The O*NET Interest Profiler, the largest operationalization of RIASEC, took more than two decades of worker surveys, expert ratings, and iterative empirical calibration to construct, and the 2022 Chinese Occupational Classification has no comparable psychological database. Large language models (LLMs) offer a scalable alternative, but using them as raters raises issues that single-model designs do not resolve: inter-rater reliability, calibration to external benchmarks, and systematic psychometric validation. We propose a multi-agent LLM framework in which three LLMs serve as separate expert raters, in-context anchors align the rating scale, and a separate arbitrator resolves rater disagreements. We applied the framework to all 1636 occupations in the 2022 Chinese Occupational Classification, producing six RIASEC and five Big Five scores per occupation. RIASEC dimensions showed uniformly excellent reliability (intraclass correlation coefficient, ICC [2,1] = 0.87 to 0.98) and high convergent correlations with O*NET (r = 0.84 to 0.96); structural validity received weak support (Tracey’s C = 0.653, ns), though the dimensions differentiated occupational categories as theory predicts, and the profile space recovered the administrative taxonomy (adjusted Rand index, ARI = 0.418). Big Five absolute agreement was uniformly high, although ICC(2,1) values for Conscientiousness and Neuroticism were attenuated by variance compression and model-level calibration offsets rather than rater disagreement. The Big Five scores are, therefore, suited to broad occupational differentiation, particularly on Openness and Extraversion, rather than to fine-grained rank ordering on Conscientiousness or Neuroticism. The framework also yields the first occupation-level RIASEC and Big Five database for the 2022 Chinese Occupational Classification, openly available for applied use.

## 1. Introduction

Occupational psychological profiling, which characterizes occupations by the psychological attributes they demand or attract, underpins career counseling, person–job matching, and workforce research. Holland’s (1997) theory of vocational personalities, which classifies occupational interests into six types (Realistic, Investigative, Artistic, Social, Enterprising, and Conventional, known collectively as RIASEC), remains the dominant framework in the field. The theory posits that individuals and work environments can be characterized by the same six-type system, and that the degree of congruence between a person’s type and their occupational environment predicts satisfaction, stability, and performance (Nauta, 2010). Over the past half-century, RIASEC has shaped the development of major career assessment instruments and guided millions of career decisions across more than 25 countries; a PsycINFO search covering 1999 to 2009 returned 2209 citations of Holland’s work alone (Nauta, 2010). The largest operationalization of this framework is the O*NET Interest Profiler, which provides RIASEC profiles for nearly 1000 U.S. occupations within the O*NET-SOC classification system (Rounds et al., 2010). Building these profiles took more than two decades. The process combined large-scale worker surveys with trained expert ratings and iterative empirical calibration that has continued since the instrument’s introduction in 1999 (Rounds et al., 2010).

The RIASEC circular structure does not generalize uniformly across cultures. A structural meta-analysis found that the circular order model was not consistently supported across U.S. ethnic-minority and international samples (Rounds & Tracey, 1996), although a subsequent study using Chinese student samples reported circular-model fit indices comparable to U.S. norms (Long et al., 2005). The structural properties of RIASEC cannot be taken for granted outside the populations in which they were originally established, and culture-specific occupational interest data remain valuable even where the broad framework transfers.

A similar pattern holds for personality. The Big Five dimensions (Openness, Conscientiousness, Extraversion, Agreeableness, and Neuroticism) provide a widely used framework for studying occupational behavior. Meta-analytic evidence consistently shows that personality traits, particularly Conscientiousness, predict job performance across occupational groups (Barrick & Mount, 1991; Barrick et al., 2001; Schmidt & Hunter, 1998), with a century of accumulated research confirming these associations (Wilmot & Ones, 2019). The Big Five structure itself has demonstrated cross-cultural replicability (McCrae & Costa, 1997), further supporting its relevance for occupational profiling across national contexts. Yet occupation-level Big Five databases have lagged far behind RIASEC resources. The most systematic effort to date is Anni et al.’s (2025) mapping of Big Five profiles for 263 ISCO-08 occupations, which drew on self-report data from 68,540 participants in the Estonian Biobank. It is the largest occupation-level personality dataset currently available, yet it covers only a fraction of the world’s occupations and is embedded in a single national and linguistic context. Both the O*NET RIASEC database and the Anni et al. (2025) Big Five database share a common constraint: they are expensive to construct and bound to the specific classification systems for which they were designed. For nations with their own occupational taxonomies, especially those outside the English-speaking world, building equivalent resources through traditional survey-based data collection remains a major undertaking, and to date, no comparable occupation-level database exists outside the O*NET and Anni et al. (2025) systems.

Large language models (LLMs) offer a scalable alternative. In natural language processing, the “LLM-as-a-judge” paradigm has gained traction as a scalable approach to evaluation, with strong LLMs achieving over 80% agreement with human preferences when judging the quality of chatbot responses, on par with the level of agreement among human evaluators themselves (Zheng et al., 2023). In psychology, researchers have explored using LLMs as simulated participants, conditioning models on demographic backstories to generate synthetic survey responses that reproduce known population-level patterns, a paradigm termed “silicon sampling” (Argyle et al., 2023; Aher et al., 2023). More directly, LLMs can perform text annotation and classification at levels matching or exceeding those of trained human coders. Gilardi et al. (2023) found that zero-shot ChatGPT (gpt-3.5-turbo) outperformed crowd workers on relevance, stance, topic, and frame detection tasks, and Törnberg (2023) reported that GPT-4 exceeded both expert coders and crowd workers in annotating political messages. These capabilities extend to multilingual settings, where GPT models outperform dictionary-based methods for psychological text analysis across 12 languages (Rathje et al., 2024). Demszky et al. (2023) provide a broad framework for integrating LLMs into psychological measurement, experimentation, and practice. Abdurahman et al. (2024), however, document cultural bias in LLM outputs and warn against treating LLMs as universal text-analysis tools in zero-shot settings.

Three methodological challenges merit closer attention when applying LLMs as raters of psychological constructs. First, much existing work relies on a single model, which leaves inter-rater reliability unestimated. Sampling a single model at varying temperatures introduces variability, but this variability is generated under one underlying conditional distribution and so does not provide the rater independence that psychometric reliability indices presume. Second, LLM rating procedures rarely anchor scores to an external measurement metric. Prompt-internal rubrics and few-shot examples can structure the rating task, but they do not align the resulting scores to an established occupational or psychological scale; without such anchoring, scores can drift across items in ways that are difficult to detect at scale. Third, while individual studies have examined the validity of LLM ratings against human benchmarks on selected criteria, comprehensive psychometric evaluations encompassing inter-rater reliability, structural validity, convergent validity, and discriminant validity within a single investigation remain rare.

Multi-agent architectures, which coordinate multiple LLMs as independent components, provide a natural solution. In natural language processing, multi-agent LLM systems have been shown to improve factual accuracy and judgment consistency through inter-model exchange, often via iterative debate (Guo et al., 2024; Du et al., 2024; Chan et al., 2024). Applied to LLM-as-rater work, this approach maps onto classical inter-rater reliability designs from psychometrics: disagreement among models becomes quantifiable, and external reference data can serve as calibration anchors paralleling the standardized training materials used in human rater certification.

The present study proposes a multi-agent LLM framework for generating occupational RIASEC and Big Five profiles and evaluates its psychometric properties. We apply the framework to all 1636 occupations in the 2022 Chinese Occupational Classification (Ministry of Human Resources and Social Security of the People’s Republic of China, 2022), which is organized under a taxonomy independent of O*NET-SOC or ISCO-08 and has no pre-existing RIASEC or Big Five database available for direct comparison. The taxonomy is, therefore, a demanding test case. It is large enough to stress-test the framework at scale and requires the framework to generate ratings without pre-existing scores to copy. Three LLMs serve as separate expert raters, O*NET RIASEC and Anni et al.’s (2025) Big Five data serve as reference anchors, and a separate LLM acts as arbitrator. We evaluate the resulting ratings through inter-rater reliability, construct validity (discriminant, structural, and convergent evidence), and sensitivity to design choices, including reference data availability and model-specific bias. Our primary contribution is methodological: a multi-agent framework in which reference anchors inform rather than dictate the rater’s judgments, evaluated through standard psychometric scrutiny. The database of psychological profiles for the 1636 occupations is a substantive byproduct.

## 2. Method

### 2.1. Occupational Data

Three occupational datasets enter the study: the target occupations to be profiled, and two reference databases that serve as calibration anchors during scoring and as external benchmarks during validation.

*Chinese occupations.* The target occupations are drawn from the 2022 edition of the Classification of Occupations of the People’s Republic of China, the current taxonomy maintained by the Ministry of Human Resources and Social Security (Ministry of Human Resources and Social Security of the People’s Republic of China, 2022). The taxonomy organizes occupations into four successively narrower levels: major categories, medium categories, minor categories, and detailed categories. The working set comprises all 1636 entries at the detailed level, distributed across all eight major categories. Each record provides a hierarchical occupation code, an occupation title, a definition paragraph, and a list of task descriptions, with task descriptions available for 1625 of the 1636 occupations.

O*NET RIASEC anchors. RIASEC reference scores come from Release 30.1 (February 2026) of the O*NET Interest Profiler. Release 30.1 covers 923 U.S. occupations under the O*NET-SOC 2019 classification and reports scores on a 1.0–7.0 scale for each of the six Holland dimensions. The Profiler is one module within the O*NET content model described by Peterson et al. (2001); the instrument itself and its scoring procedure are documented in the O*NET Interest Profiler technical manual (Rounds et al., 2010). These scores serve two roles. First, during scoring, they are injected into the expert prompt as calibration anchors that align the agents’ score distributions with a reference metric. Second, during evaluation, they serve as the external benchmark against which convergent validity is estimated for RIASEC.

*Anni et al. Big Five anchors.* Big Five reference scores are derived from the Smoothed Residualized Personality Scores in Table S14 of Anni et al. (2025). These scores cover 263 ISCO-08 occupations and are based on self-reports from 68,540 respondents in the Estonian Biobank. The source scores use a *T* metric (*M* = 50, *SD* = 10) anchored to the Estonian general population. To rescale onto the Chinese working-adult Big Five Inventory-2 metric (1–5; BFI-2 instrument: Soto & John, 2017), we used the Chinese employee-sample norms (*n* = 486) reported in Table 1 of Zhang et al. (2022). The conversion proceeds dimensionwise: first *Z* = (*T* − 50)/10, then the China-scaled value $S\;=\;Z\;\times \;SD_{China}\;+\;M_{China}$, where $M_{China}$ and $SD_{China}$ are the dimension-specific values from these norms (Supplementary Material S4.1 provides the full norm table).

*Crosswalks.* The RIASEC anchors are indexed under O*NET-SOC 2019 and the Big Five anchors under ISCO-08, so anchor retrieval requires a short chain of crosswalks. O*NET-SOC 2019 codes are linked to O*NET-SOC 2010 through the official O*NET version crosswalk. They are then linked to ISCO-08 through a compiled direct mapping derived from the SOC-to-ISCO crosswalk of Hardy et al. (2018). After consolidation, 998 of the 1012 O*NET-SOC 2019 codes map to at least one ISCO-08 code.

### 2.2. Multi-Agent Architecture

**Figure 1:**
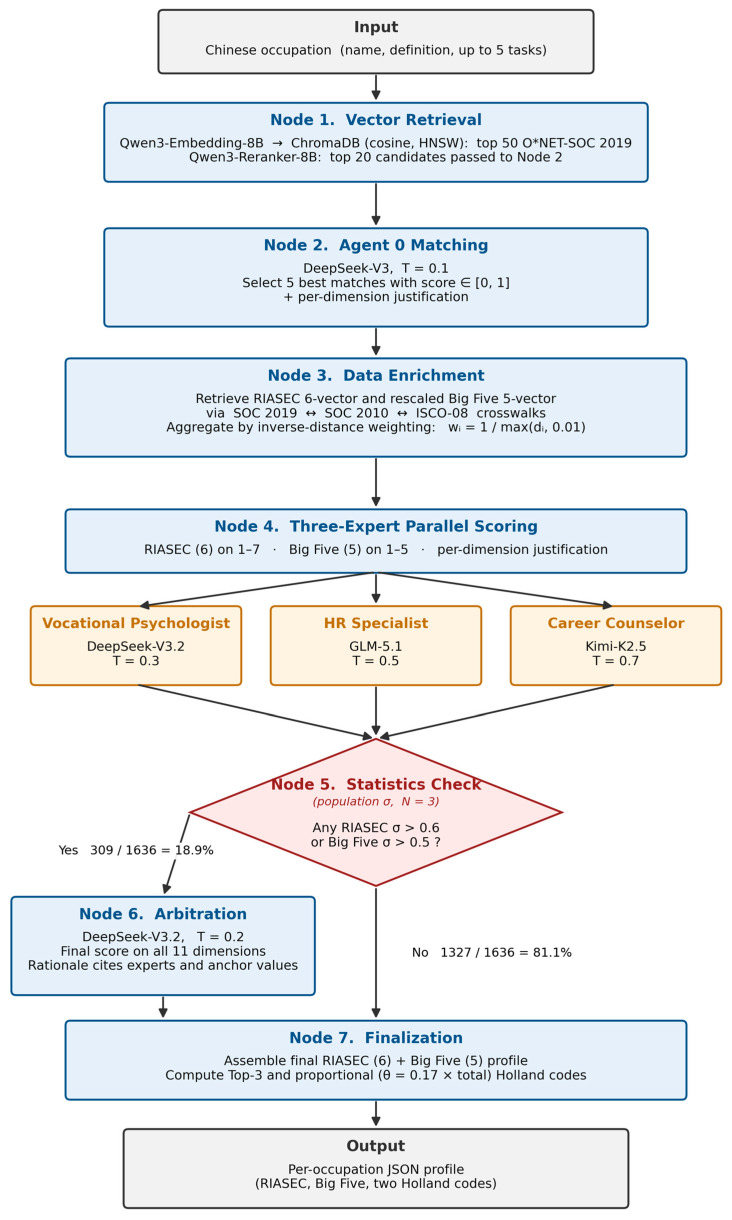
The seven-node multi-agent workflow for occupational profile generation. Note: The diagram traces the seven-node directed workflow implemented in LangGraph for one target occupation. Node 1 retrieves O*NET candidates by vector similarity (Qwen3-Embedding-8B + Qwen3-Reranker-8B); Node 2 selects the five best matches via the Agent 0 matcher (DeepSeek-V3, *T* = 0.1); Node 3 aggregates RIASEC and Big Five reference values into anchors through inverse-distance weighting; Node 4 runs three expert LLMs in parallel (DeepSeek-V3.2 at *T* = 0.3, GLM-5.1 at *T* = 0.5, Kimi-K2.5 at *T* = 0.7); Node 5 computes between-rater dispersion and flags any RIASEC dimension with σ > 0.6 or any Big Five dimension with σ > 0.5; Node 6 (conditional, triggered for 309 of the 1636 occupations or 18.9%) invokes a separate arbitrator (DeepSeek-V3.2, *T* = 0.2); Node 7 assembles the final profile and assigns Holland high-point codes. Solid arrows denote the default path; the dashed arrow denotes the conditional arbitration branch.

Profile generation is implemented as a seven-node directed workflow in LangGraph (v1.1.6), a framework for composing stateful LLM pipelines. A fixed directed workflow, rather than a self-directed (e.g., ReAct) agent, is deliberate. The unit of analysis is a psychometric rating, so every occupation must pass through identical processing for the reliability and validity indices to be well defined. A fixed graph guarantees this: the three expert raters score the same input in parallel without cross-talk, preserving their independence, and the path is fully auditable and reproducible. For each target occupation, the system traverses the nodes summarized in Figure 1. A shared state object carries the occupation input and the intermediate outputs across nodes. All LLM calls use the SiliconFlow endpoint, a structured JSON response format, a 180 s timeout, and up to three attempts with exponential backoff. Retries are triggered by transient network errors and by HTTP 5xx or 429 responses.

*Retrieval and matching.* Node 1 embeds the target occupation title with the Qwen3-Embedding-8B model. It then queries a persistent ChromaDB index in which all O*NET-SOC 2019 occupations have been pre-indexed using cosine-similarity HNSW. The top 50 candidates are retrieved and then re-ranked against the full query text with the Qwen3-Reranker-8B model, retaining the top 20. Node 2 passes these 20 candidates, together with the Chinese occupation name, definition, and up to five task descriptions, to an Agent 0 matcher (DeepSeek-V3, *T* = 0.1). Agent 0 is instructed to select the five candidates closest to the Chinese occupation in terms of work content, skill requirements, knowledge domain, work environment, and occupational level. Each selection is returned with a match score in [0, 1] and a per-dimension justification.

*Data enrichment.* For each of the five matched O*NET codes, Node 3 retrieves the RIASEC six-vector from the Interest Profiler table. Via the crosswalks, it also retrieves the corresponding ISCO-08 code and its rescaled Big Five five-vector. The five candidates are aggregated into a single anchor through inverse-distance weighting. For each candidate *i*, a raw weight $w_{i}\;=\;1/max(d_{i},\;0.01)$ is computed from its cosine vector distance $d_{i}$. The weights are normalized to sum to one and then applied dimensionwise. RIASEC and Big Five are normalized independently. The individual candidate scores and the aggregated weighted means are both carried forward in the state.

*Three-expert parallel scoring.* Node 4 runs three expert agents concurrently on the same input and the same anchor payload. The three agents differ in the underlying LLM and in the role description supplied through the system message. They are a vocational psychologist backed by DeepSeek-V3.2, a human-resources specialist backed by GLM-5.1, and a career counselor backed by Kimi-K2.5, all served through SiliconFlow. Each agent returns scores on all six RIASEC dimensions (clamped to 1–7) and on all five Big Five dimensions (clamped to 1–5). Each score is accompanied by a brief per-dimension justification and a free-text summary. Each expert agent was configured with a distinct temperature (*T* = 0.3, 0.5, and 0.7, respectively) to further diversify response characteristics. The matcher (*T* = 0.1) and arbitrator (*T* = 0.2) instead run near-deterministically, since selection and adjudication call for stability rather than diversity. Four considerations drove the choice of models. First, the occupations are defined in Chinese, so we chose models with strong native Chinese comprehension to read the official definitions and tasks without translation loss. Second, all three are open-weight models from different developers and pretraining pipelines: the different origins widen rater heterogeneity and reduce, though do not remove, common-source dependence, and because the weights are openly released, the exact model versions remain available for independent replication, which closed commercial models do not allow. Third, all three were current, strong-performing models when scoring was run, so the design did not trade away capability. Fourth, all three are reachable through one SiliconFlow endpoint, which kept scoring 1636 occupations across three raters and an arbitrator tractable in cost and engineering.

The scoring prompt is organized into four blocks that are shared across the three agents and one block that differentiates their evaluative stance. The shared blocks cover the target occupation definition and tasks, the reference anchor payload described in Section 2.3, the dimensional descriptors and their verbal anchors, and the required response schema. The central instruction is stated in the system message and illustrated with worked examples: each agent must rate the *ideal* worker for the occupation, not the *typical* current incumbent. The neuroticism scale is additionally annotated to clarify the distinction between trait Neuroticism (emotional reactivity in the worker) and occupational stress (in the work environment), in line with the ideal-worker frame. The role-specific block focuses each agent’s attention on a different facet of the occupation. The vocational psychologist foregrounds theory-driven task-to-trait mapping; the human-resources specialist foregrounds competency-model and work-environment fit; the career counselor foregrounds long-run person–job congruence and adherence to anchor values.

*Statistics check and arbitration.* Node 5 pools the three expert ratings into dimensionwise means and population standard deviations (dividing by *N* = 3). An occupation is flagged for arbitration whenever any RIASEC dimension has σ > 0.6 or any Big Five dimension has σ > 0.5. The two thresholds are static operational settings, chosen by design convention to produce a comparable arbitration-triggering base rate across the two systems. The two values correspond to 10% of the 1–7 RIASEC range and 12.5% of the 1–5 Big Five range. Node 5 also assigns each dimension a confidence label (high, medium, or low) derived from σ for the per-occupation report. If any dimension triggers the arbitration flag, Node 6 invokes a separate arbitration agent backed by DeepSeek-V3.2 at *T* = 0.2. The arbitrator receives the occupation definition, the anchor payload, each expert’s full score and justification, and a summary of the disputed dimensions. It then issues a final score for all 11 dimensions, together with an explicit rationale that cites specific experts and anchor values. Missing dimensions in the arbitrator’s output are back-filled with the corresponding expert mean, and all returned scores are clamped to their valid range.

*Finalization.* Node 7 assembles the final profile from whichever score source applies (the expert mean or the arbitration verdict). It also records two Holland high-point codes. The first is a standard Top-3 code formed from the three highest RIASEC dimensions in descending order. The second is a proportional high-point code that retains up to three dimensions scoring at or above 0.17 × total. The 0.17 cutoff is the threshold adopted by the O*NET Occupational Interest Profile procedure for assigning second- and third-letter Holland codes (Rounds et al., 1999), corresponding to the equal-share baseline of 1/6: if the six Holland dimensions contributed equally to the profile, each would account for roughly this share of the total. Dimensions above this baseline contribute more than an equal share and are retained; the retained set is truncated to the top three in descending order.

The Agent 0 matcher prompt and the full system messages of the three expert agents and the arbitrator are reproduced verbatim in Supplementary Material S1 (S1.1 for the matcher, S1.3 for the expert agents, and S1.4 for the arbitrator). The same supplement includes the shared user message template, the worked examples, the dimensional rubrics, and the response schemas. A worked example showing the full node-by-node trace is provided in Supplementary Material S2. The example covers a non-arbitrated occupation (*Psychological Counselor*, 2-07-10-03) and an arbitrated occupation (*Director*, 2-09-01-06), with all intermediate outputs between Node 1 and Node 7 reproduced from the per-occupation JSON records.

### 2.3. Reference Anchoring

The anchor payload is the mechanism through which external psychometric data enter the scoring decision. For each target occupation, the prompt includes a reference-anchors block listing the five matched O*NET occupations and their RIASEC and (when available) rescaled Big Five vectors, together with inverse-distance-weighted means. The full prompt template is reproduced in Supplementary Material S1.2.

The prompt frames these anchors as calibration reference points rather than targets to reproduce. It notes that the reference data originate in different cultural and linguistic contexts, asks each rater to derive the rating from the Chinese occupational definition independently, and requires that any deviation from the anchor be explained in the per-dimension justification. The pipeline neither averages the anchor values into the final score nor imposes them as hard constraints.

The retrieval-plus-crosswalk pipeline produces three anchor-coverage strata (full anchor, RIASEC-only, no anchor) used for the sensitivity analysis in Section 3.6.

### 2.4. Psychometric Evaluation Plan

The ratings are evaluated through four classes of psychometric evidence that bear directly on profile-level validity: inter-rater reliability, discriminant validity, structural validity, and external convergent validity. A fifth class of mechanism analyses (sensitivity and model-bias) probes the internal drivers of the first four.

*Inter-rater reliability.* Treating the three expert models as separate raters, we estimate two-way random-effects intraclass correlation coefficients ICC(2,1) and ICC(2,*k*) for absolute agreement. We additionally estimate the two-way mixed-effects ICC(3,1) for consistency after rater main effects are removed (Shrout & Fleiss, 1979; Koo & Li, 2016). ICC(2,1) serves as the primary reliability index because it penalizes both systematic and random between-rater differences; ICC(3,1) serves as the diagnostic index for rater main effects (interpretation of the gap between the two is taken up in Section 3.6 and Section 4.2). ICC is complemented with the mean absolute deviation (MAD) across raters within each occupation and dimension, an absolute-agreement index that is not attenuated by between-occupation variance restriction. Holland high-point agreement is additionally reported as the first-letter match rate and the unordered top-two match rate across the three experts.

*Discriminant validity.* A multitrait–multimethod (MTMM) matrix is constructed with the six RIASEC dimensions as traits and the three expert LLMs as methods (Campbell & Fiske, 1959). This yields an 18 × 18 correlation matrix. The block structure separates three classes of correlations: monotrait-heteromethod correlations measure convergent agreement on the same dimension across different raters; heterotrait-monomethod correlations measure how sharply a single rater distinguishes between dimensions; and heterotrait-heteromethod correlations measure incidental covariation across both trait and rater.

Convergent validity is evaluated by whether the monotrait-heteromethod diagonal is positive. Discriminant validity is evaluated through the four Campbell-and-Fiske criteria in turn: the monotrait-heteromethod values exceed zero, exceed the corresponding heterotrait-heteromethod off-diagonals, exceed the heterotrait-monomethod within-rater off-diagonals, and preserve their rank order of traits across different method blocks. We summarize each block of correlations with its central tendency and report the proportion of Campbell-and-Fiske criteria that are satisfied.

The MTMM matrix was confined to the six RIASEC dimensions. A separate Big Five MTMM was not constructed because Conscientiousness and Neuroticism are expected to exhibit restricted between-occupation variance under the ideal-worker rating frame, since most occupations require an emotionally stable and conscientious incumbent. Variance restriction attenuates monotrait-heteromethod correlations independently of actual rater agreement, rendering Campbell-and-Fiske criteria uninformative on these two dimensions regardless of whether the Big Five are evaluated alone or alongside RIASEC. Between-rater agreement on the Big Five is, therefore, evaluated through ICC, MAD, and model-bias analyses, which are less sensitive to between-target variance restriction.

*Structural validity.* For RIASEC, the circular structure is tested with the randomization test of hypothesized order relations (Hubert & Arabie, 1987), applied to Holland’s circular model following Rounds and Tracey (1996), and implemented from first principles in R with 10,000 permutations. We report the correspondence index and its randomization *p* value. As an auxiliary check, we inspect the ordered pattern of adjacent, alternating, and opposite correlations. A set of known-group ANOVAs then tests whether each RIASEC dimension peaks on its theoretically indicated occupational category, derived from Holland’s (1997) RIASEC environment-type framework (e.g., Realistic environments correspond to manufacturing and agriculture; Conventional environments correspond to clerical work). Predictions are formulated for the major groups whose Holland-environment mapping is unambiguous; the Service and Others-Unclassifiable major groups, which span multiple Holland environments, are not the target of a priori predictions but enter the omnibus ANOVAs as additional levels.

For Big Five, the factor intercorrelation matrix at the occupation level is compared against four sign patterns from the individual-level Big Five literature (van der Linden et al., 2010): negative Neuroticism–Conscientiousness, negative Neuroticism–Agreeableness, positive Extraversion–Agreeableness, and positive Agreeableness–Conscientiousness.

*Convergent validity.* Convergent validity is estimated as the dimensionwise correlation between the final profiles and two external reference sets: the O*NET RIASEC scores for the RIASEC dimensions, and the rescaled Anni et al. (2025) Big Five scores for the Big Five dimensions. The occupation-level match is generated by the crosswalks in Section 2.1. These reference sets also serve as calibration anchors during scoring; auxiliary tests of anchor independence are reported in Section 3.4 and Section 3.6. Holland high-point match rates against the O*NET benchmark are reported as a complementary index for RIASEC.

*Sensitivity and model-bias analyses.* A sensitivity analysis compares score distributions, reliability, and anchor deviation across the three anchor-coverage strata defined in Section 2.3. This assesses the degree to which observed psychometric properties are carried by the anchors versus the agents’ independent judgments. Because the strata also differ in occupational composition, we additionally isolated the contribution of the anchors with a within-occupation experiment: a stratified random subsample of 200 occupations drawn from the full-anchor stratum was re-scored under three masked anchor conditions (RIASEC anchors only, Big Five anchors only, and no anchors), using the same models, prompts, and thresholds, with the full-anchor scores as the baseline. Because the four conditions share occupations, anchor effects are estimated within occupation and are not confounded with occupational content. For each dimension, we compared each masked condition against the full-anchor baseline with a paired-samples *t*-test and Cohen’s *d*, and recomputed ICC(2,1) within each condition.

A model-bias analysis then probes whether any single LLM systematically shifts ratings relative to the other two. For each of the 11 dimensions, we run a one-way repeated-measures ANOVA on the raters’ scores. The within-occupation factor is a rater model (three levels). Occupation is the repeated-measures unit. The main effect of a rater model on a given dimension tests whether the three LLMs differ in overall mean on that dimension. Pairwise post hoc contrasts (Bonferroni-corrected paired *t*-tests) localize which pair of models drives any significant effect, with Hedges’ *g* reported for each contrast. A systematic dimension-level shift from a single model is read from two signatures. The first is a non-negligible partial η^2^ on that dimension. The second is a consistent sign on the two pairwise *g* values involving that model. The two analyses address complementary dimensions of residual variance: the sensitivity analysis covers anchor coverage, and the model-bias analysis covers rater identity. The two sources of systematic variation can, therefore, be attributed separately rather than conflated.

*Software.* The multi-agent pipeline (Section 2.2 and Section 2.3) was implemented in Python 3.11 with LangGraph for workflow orchestration; per-occupation profile assembly and the descriptive overview (Section 3.1) used pandas. All inferential analyses reported in Section 3.2, Section 3.3, Section 3.4, Section 3.5 and Section 3.6 were conducted in R 4.5.3 (R Core Team, 2026), with intraclass correlation coefficients computed in the psych package (Revelle, 2025) and cross-checked in irr (Gamer et al., 2019), repeated-measures ANOVAs in afex (Singmann et al., 2024), marginal means and Bonferroni-corrected pairwise contrasts in emmeans (Lenth, 2024), and partial η^2^ with 90% confidence intervals and paired Hedges’ *g* in effectsize (Ben-Shachar et al., 2020) and effsize (Torchiano, 2020). The *k*-means cluster analysis (Supplementary Material S3.1) and the per-occupation reference-coverage stratification (Section 3.6) used Python with scikit-learn. All R and Python code, raw outputs, and the per-occupation profile dataset are publicly available at https://osf.io/gdjb4/ (scored on 10 April 2026; versions in Supplementary Material S1).

*Reproducibility.* The framework is fully specified for conceptual reproduction: the workflow (Section 2.2), the prompts (Supplementary Material S1), the thresholds, and the analysis code (OSF) let the pipeline be rebuilt and rerun. Exact numerical reproduction is bounded by the serving endpoint, not by the models: we accessed them through SiliconFlow, whose hosted snapshots may change or be retired and are not fully deterministic even at low temperature. Because the three models are open-weight, the exact versions remain publicly available, so the pipeline can be re-hosted and reproduced more faithfully than closed-API systems allow. We record the model identifiers served by SiliconFlow, release every prompt verbatim, and provide the complete per-occupation outputs on OSF, so every reported analysis can be reproduced from the released scores without rerunning the models. Users who rescore against newer models should expect small shifts and re-anchor and re-validate accordingly.

## 3. Results

The framework was applied to all 1636 occupations in the 2022 Chinese Occupational Classification. The five evaluation classes outlined in Section 2.4 are reported in Section 3.2, Section 3.3, Section 3.4, Section 3.5 and Section 3.6.

### 3.1. Descriptive Overview

The pipeline produced complete RIASEC and Big Five profiles for all 1636 target occupations with no missing cases at output. Arbitration was triggered for 309 occupations (18.9%); the remaining 1327 (81.1%) were finalized at the expert-mean stage. Retrieval-based O*NET matching achieved a mean match score of 0.901 (SD = 0.047) across the five top candidates per occupation, indicating close counterparts in the O*NET-SOC 2019 catalog. At the occupation level, 1310 cases (80.1%) received full RIASEC and Big Five anchors, 318 (19.4%) received RIASEC anchors only, and 8 (0.5%) received no anchor.

**Figure 2:**
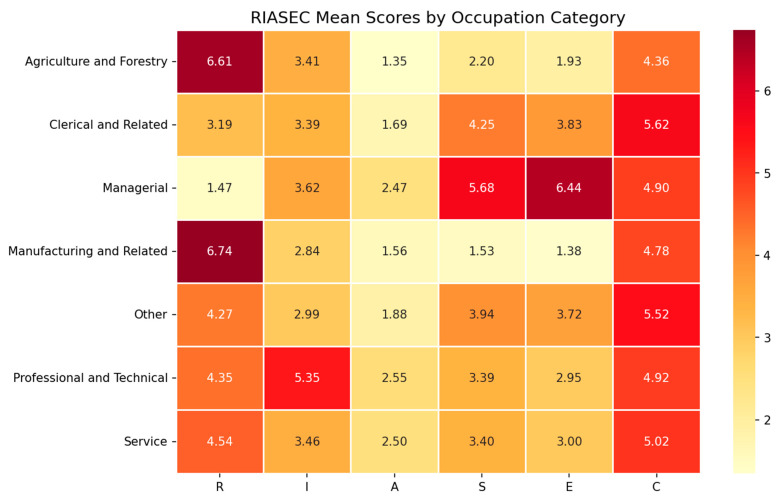
Major-Category × RIASEC heatmap. Note: Cells show mean RIASEC scores (1–7 scale) within each of seven major occupational categories of the 2022 Chinese Occupational Classification; darker cells mark higher values. *n* = 1636 occupations.

The RIASEC descriptive statistics (Table 1) were consistent with a classification weighted toward production and technical occupations. Realistic scored highest (*M* = 5.37, *SD* = 1.85) and Artistic lowest (*M* = 2.07, *SD* = 1.41); Conventional (*M* = 4.89, *SD* = 0.77) had the second-highest mean and the narrowest dispersion. Skewness was strongly negative for Realistic (−1.01) and strongly positive for Artistic (2.00), matching a taxonomy that tilts toward hands-on occupations. Holland first-letter codes were dominated by Realistic (*n* = 939, 57.4%), followed by Investigative (*n* = 244, 14.9%) and Conventional (*n* = 181, 11.1%); the most frequent three-letter code, RCI, accounted for 701 occupations (42.9%). Figure 2 summarizes the Major-Category × RIASEC distribution.

Big Five descriptive statistics (Table 2) showed small raw standard deviations on two dimensions. Conscientiousness had the highest mean (*M* = 4.43, *SD* = 0.21) and Neuroticism the lowest (*M* = 1.85, *SD* = 0.19). The Conscientiousness and Neuroticism *SD*s corresponded to 5.2% and 4.7% of the four-unit scale range (1–5), respectively. These values aligned with the ideal-worker rating frame described in Section 2.2.

### 3.2. Inter-Rater Reliability

We evaluated inter-rater reliability on the three expert agents’ independent scores prior to arbitration. Ten occupations returned only two of three expert scores and were excluded from the ICC pool; no occupation was fully missing. The reliability sample, therefore, comprised 1626 occupations with complete triplets. Table 3 reports ICC indices, mean absolute deviations, and Holland high-point agreement rates.

*Absolute agreement.* Two-way random-effects absolute-agreement ICCs for single raters were high across RIASEC. Values ranged from 0.866, 95% CI [0.85, 0.88] for Conventional to 0.979 [0.98, 0.98] for Realistic, with ICC(2,*k*) from 0.951 to 0.993. All six dimensions satisfied the conventional excellent-reliability threshold for a single rater (Cicchetti, 1994). Big Five ICCs were more dispersed. Openness (ICC [2,1] = 0.871, 95% CI [0.86, 0.88]) and Extraversion (0.842 [0.79, 0.88]) reached the good-to-excellent range; Agreeableness (0.678 [0.48, 0.79]) was in the good range; Conscientiousness (0.522 [0.34, 0.65]) and Neuroticism (0.374 [0.12, 0.56]) fell into the fair-to-poor range. ICC(2,*k*) aggregated to 0.864 for Agreeableness, 0.766 for Conscientiousness, and 0.642 for Neuroticism when the three raters were combined.

*Mean absolute deviation.* The low Big Five ICCs co-occurred with MAD values among the smallest in the full 11-dimension set. On the 1–5 Big Five scale, Conscientiousness had the lowest MAD (*M* = 0.115, 2.9% of the scale), followed by Neuroticism (*M* = 0.142, 3.6%). The six RIASEC MADs ranged from 0.137 for Realistic (2.3% of the 1–7 scale) to 0.257 for Investigative (4.3%). The within-to-between standard-deviation ratio $\frac{\sigma_{w}}{\sigma_{b}}$ explains the pattern: low ICC alongside low MAD indicates restricted between-occupation variance rather than large within-occupation disagreement. For Realistic, the ratio was 0.100, indicating agreement dominated by between-occupation variation. For Conscientiousness, it was 0.739, and for Neuroticism, 1.025, so within-occupation disagreement was comparable to or exceeded between-occupation variation (complete ratios for all 11 dimensions in Table S3.3 of Supplementary Material S3). The consistency analysis below identifies a second contributing factor.

*Consistency after removing rater main effects.* Comparing ICC(3,1), which removes rater main effects, with ICC(2,1) isolates the contribution of systematic between-rater differences (Table 3). For RIASEC, the two indices were nearly identical (Δ ≤ 0.024 across the six dimensions), indicating negligible rater main effects. For Big Five, the gaps were larger. Neuroticism increased from 0.374 to 0.540 (Δ = 0.166), Agreeableness from 0.678 to 0.763 (Δ = 0.084), and Conscientiousness from 0.522 to 0.611 (Δ = 0.089); Openness and Extraversion changed by less than 0.020. Together with the MAD analysis, these results indicate that the lower Big Five ICC(2,1) values are jointly produced by restricted between-occupation variance and systematic model-level calibration shifts on Neuroticism, Agreeableness, and Conscientiousness. Even after the mean shifts are removed, the ICC(3,1) values show good rank-order agreement for Agreeableness but only moderate agreement for Conscientiousness and fair agreement for Neuroticism.

*Holland high-point agreement.* Across the 1626 occupations with complete triplets, the three experts agreed on the first Holland letter for 83.5% and on the unordered top two letters for 78.8%; the ordered top two letters matched for 67.5%. The 11.3-percentage-point gap between unordered and ordered top-two agreement indicates that the main source of categorical mismatch is disagreement about which of the top two letters comes first, not which two letters qualify.

### 3.3. Convergent and Discriminant Evidence from the Multitrait–Multimethod Matrix

We assessed convergent and discriminant evidence at the rater level with a multitrait–multimethod matrix treating the six RIASEC dimensions as traits and the three expert LLMs as methods (Campbell & Fiske, 1959). The resulting 18 × 18 correlation matrix spanned 1626 occupations. Because the three methods are LLMs with partly overlapping pretraining data and a shared prompt and anchor payload, monotrait-heteromethod convergence here indexes agreement across models, not accuracy against an external criterion.

Monotrait-heteromethod correlations, which index agreement on the same trait across different raters, were high (*n* = 18; *M* = 0.949, *Mdn* = 0.963, range 0.850 to 0.983). The 18 coefficients exceeded zero in every case, satisfying Campbell and Fiske’s first criterion. The full 18 × 18 correlation matrix is reproduced in Supplementary Material S4. The 90 heterotrait-heteromethod correlations, which index incidental covariation across both trait and rater, averaged near zero (*M* = −0.060, *Mdn* = 0.026, range −0.812 to 0.670). The difference between the two blocks was large (Δ = 1.01, Cohen’s *d* = 3.64), satisfying their second criterion. The 45 heterotrait-monomethod correlations also centered near zero (*M* = −0.057), so trait separation was not artifactually inflated within a single method.

**Figure 3:**
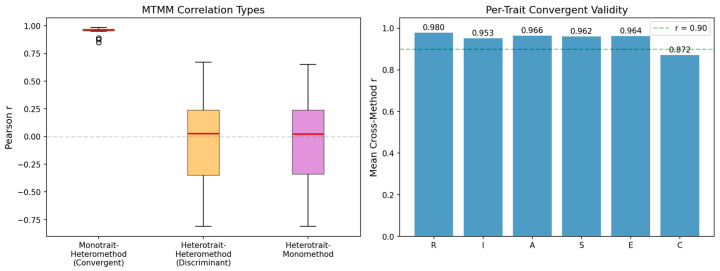
MTMM monotrait-heteromethod vs. heterotrait-heteromethod block separation. Note: Left panel shows box-whisker distributions of the three MTMM correlation blocks: monotrait-heteromethod (convergent; *n* = 18), heterotrait-heteromethod (discriminant; *n* = 90), and heterotrait-monomethod (within-method; *n* = 45). Boxes show the IQR, red lines the median, whiskers the 1.5 × IQR range, and circles the outliers on the convergent block. Right panel shows mean cross-method *r* per trait; the green dashed line marks *r* = 0.90 as a descriptive reference. R = Realistic; I = Investigative; A = Artistic; S = Social; E = Enterprising; C = Conventional.

Averaging the monotrait-heteromethod coefficients by trait yielded an ordering that closely paralleled the dimension-level ICCs in Section 3.2, with Artistic and Enterprising swapping adjacent positions. Agreement was highest for Realistic (mean *r* = 0.980), followed by Artistic (0.966), Enterprising (0.964), Social (0.962), and Investigative (0.953); Conventional was lowest (0.872), in line with its restricted between-occupation variance. Figure 3 illustrates the separation between the convergent and discriminant blocks. Campbell and Fiske’s first two criteria (convergent correlations above zero and convergent correlations exceeding discriminant correlations) were satisfied numerically, and the heterotrait pattern was consistent across the three methods (cell-level verification of all four criteria in Supplementary Material S4).

### 3.4. Structural Validity

**Figure 4:**
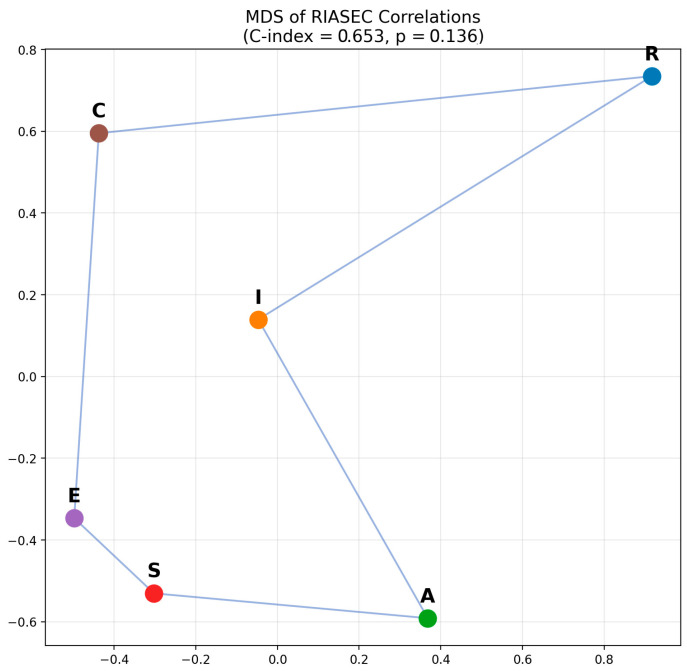
Multidimensional scaling configuration of the six RIASEC dimensions across 1636 Chinese occupations. Note: R = Realistic; I = Investigative; A = Artistic; S = Social; E = Enterprising; C = Conventional. Points plot the six RIASEC dimensions in a two-dimensional MDS solution of the 6 × 6 correlation matrix; connecting lines trace the theoretical hexagonal order R-I-A-S-E-C. Investigative sits toward the configuration centroid rather than on the outer perimeter, which accounts for the subthreshold *C*-index.

*RIASEC circular structure.* The randomization test of hypothesized order relations (Hubert & Arabie, 1987), implemented in R with 10,000 permutations following the procedure of Rounds and Tracey (1996), returned a correspondence index of *C* = 0.653 and a randomization *p* value of 0.136. The *p* is nonsignificant. The *C* value fell below the empirical U.S. benchmark of approximately 0.70 reported for Holland’s circular order model across U.S. interest-inventory samples (Tracey & Rounds, 1993; Rounds & Tracey, 1996). The gradient, however, was ordinally correct: adjacent mean *r* = 0.094, alternating mean *r* = −0.077, opposite mean *r* = −0.333. Figure 4 shows the six dimensions arranged in a broadly circular multidimensional scaling (MDS) configuration that approximates the theoretical antipodal pairs (Realistic–Social, Investigative–Enterprising, and Artistic–Conventional). The subthreshold C value, therefore, reflects magnitude-level attenuation of the predicted gradient rather than a reversal of the ordinal structure.

*Known-group effects.* Of the eight major categories of the 2022 Chinese Occupational Classification, only the Military category lacked detailed entries and was excluded (k = 7, N = 1636). All seven remaining categories entered the omnibus ANOVAs as factor levels. We formulated six category-by-dimension predictions (Table 4) covering the five categories with unambiguous Holland-environment mapping. Managerial receives two predictions (Enterprising and Social), with the Social prediction assigned there in the absence of a separate education/healthcare major category. All six predicted rankings were observed. Across the four dimensions with the strongest effects (Realistic, Investigative, Social, and Enterprising), partial $\eta^{2}$ ranged from 0.454 to 0.476 (90% CIs spanning 0.426 to 0.500), F(6, 1629) > 225, p < 0.001. Artistic, for which no a priori prediction was formulated (the Chinese taxonomy has no artistic-environment major category), was smaller but significant ($\eta^{2}\;=\;0.120,\;90\%\;\mathrm{CI}\;[0.095,\;0.143],\;F(6,\;1629)\;=\;37.14$). Conventional showed the predicted ranking (clerical occupations scored highest) but with a substantially smaller effect ($\eta^{2}\;=\;0.051,\;90\%\;\mathrm{CI}\;[0.033,\;0.067],\;F(6,\;1629)\;=\;14.70,\;p\;<\;0.001$).

Big Five $\eta^{2}$ values ranged from 0.147, 90% CI [0.120, 0.171], for Conscientiousness to 0.543, 90% CI [0.518, 0.565], for Extraversion, F(6, 1629) > 46, p < 0.001. A complementary cluster analysis (k = 5, silhouette = 0.305) confirmed moderate overlap between the 11-dimension groupings and the administrative taxonomy (adjusted Rand index, ARI = 0.418; see Supplementary Material S3.1). The five clusters spanned production and manufacturing, technical research, managerial service, social service, and artistic and creative groupings.

*Big Five intercorrelations.* The factor intercorrelation matrix (Table 5) matched the expected occupation-level sign pattern on two of four reference contrasts. Neuroticism correlated negatively with Conscientiousness (r = −0.76), and Extraversion correlated positively with Agreeableness (r = 0.73); both matched meta-analytic expectations. The Neuroticism–Agreeableness correlation (r = 0.48) and the Agreeableness–Conscientiousness correlation (r = −0.43) reversed the sign observed at the individual level in meta-analyses, a mismatch we return to in the Discussion section.

### 3.5. Convergent Validity

We estimated convergent validity against two reference benchmarks: the O*NET Interest Profiler for RIASEC and Anni et al. (2025) for Big Five. Matches to Chinese occupations used the Section 2.1 crosswalks, and Table 6 and Table 7 report the dimensionwise correlations. All 95% CIs for r were computed via the Fisher r-to-z transformation. Unlike the anchor payload in Section 2.3, which inverse-distance-weights across the top five matches, the convergent-validity step compares each Chinese occupation to a single best-match benchmark entry. The effective sample is, therefore, smaller than the anchor-coverage counts in Section 3.1. Because the same O*NET and Anni et al. data also served as scoring anchors (Section 2.3), the two benchmarks are only partially independent of the profiles they evaluate. The correlations below are, therefore, best read as an upper bound on convergent agreement.

**Figure 5:**
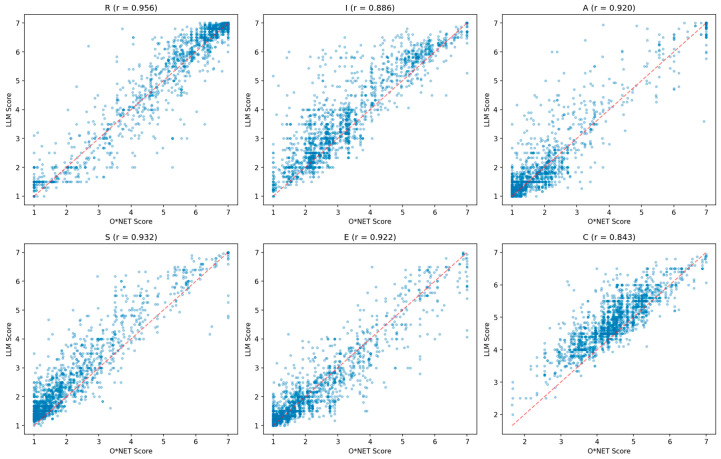
LLM vs. O*NET RIASEC scatter by dimension. Note: Each panel plots LLM-generated RIASEC scores (y) against matched O*NET Interest Profiler scores (x) on a 1–7 scale across the 1552 occupations with at least one matched O*NET entry; the red dashed line marks y = x. Panel-title r values correspond exactly to the Table 6 coefficients.

RIASEC profiles against O*NET. The crosswalk yielded 1552 occupations with at least one matched O*NET entry. Dimensionwise Pearson correlations with the O*NET RIASEC vector were high across all six dimensions (*r* = 0.843–0.956, all *p* < 0.001; see Table 6). The pooled correlation across 1552 × 6 occupation-dimension pairs was r = 0.952, 95% CI [0.950, 0.954]. Holland first-letter match rates against the O*NET benchmark reached 83.8% at the occupation level. LLM-generated scores were systematically higher than matched O*NET values on three dimensions, with mean differences of 0.44 for Social, 0.43 for Investigative, and 0.42 for Conventional on the 1–7 scale; shifts for Realistic (0.07), Artistic (0.14), and Enterprising (−0.03) were much smaller. Figure 5 shows that these positive shifts preserved rank order.

*Big Five profiles against Anni et al*. The crosswalk from O*NET-SOC 2019 to ISCO-08 yielded 798 occupations matched to at least one Anni et al. entry. Dimensionwise Pearson correlations with the rescaled Anni et al. Big Five vector ranged from 0.245 to 0.740, all *p* < 0.001 (Table 7). Convergence was highest for Openness (*r* = 0.740) and Extraversion (*r* = 0.640), and lower for Conscientiousness (*r* = 0.372), Neuroticism (*r* = 0.344), and Agreeableness (*r* = 0.245). The pooled correlation across 798 × 5 occupation-dimension pairs was *r* = 0.859, 95% CI [0.851, 0.867]. The two strongest convergent dimensions (Openness and Extraversion) corresponded to the two Big Five dimensions with the largest between-occupation variance in the present data (*SD* = 0.65 and 0.56, respectively); the three weakest clustered at the low-variance end. The within-cluster ordering was not strictly monotonic: Agreeableness showed the lowest r (0.245) despite holding the third-largest SD (0.43), so variance restriction was not the sole determinant of convergent strength at the dimension level. Mean differences against the Anni et al. benchmark were small for Openness (0.07), substantial for Conscientiousness (+0.69) and Neuroticism (−0.79), and smaller for Extraversion (−0.17) and Agreeableness (−0.19). LLM-generated profiles thus produced systematically higher Conscientiousness and lower Neuroticism scores than the self-report-based Anni et al. reference. This directional offset aligned with the ideal-worker framing in Section 2.2: the agents rated a successful incumbent rather than a typical respondent, so nearly every ideal profile is high in Conscientiousness and low in Neuroticism. The resulting compression of between-occupation variance (Section 3.1) produces a systematic gap against Anni et al.’s self-report baseline that is independent of rank-order agreement.

### 3.6. Sensitivity and Bias

*Anchor coverage.* The primary contrast lies between the full-anchor (*n* = 1310) and RIASEC-only (*n* = 318) strata; the no-anchor stratum (*n* = 8) is too small for stable comparisons. Between-stratum differences in Big Five distributions and reliability were substantial (Table 8): Cohen’s *d* ranged from 1.54 to 2.11 in absolute value across the five dimensions, and ICC(2,1) dropped sharply in the RIASEC-only group (e.g., Openness from 0.839 to 0.586; Neuroticism from 0.284 to 0.094). These effect sizes are inflated by the compressed within-stratum variance of the RIASEC-only group (Big Five SDs of 0.09 to 0.35 vs. 0.17 to 0.55 in the full-anchor group; Table 8). Because Cohen’s *d* scales by the pooled *SD*, restricted variance enlarges *d* independently of the raw mean gap (Δ*M* ≤ 0.97 on the 1–5 scale), and the large stratum sizes (*n* = 1310 vs. 318) make the *t* values trivially large. The raw mean difference (Δ*M*) is the more interpretable index. The anchor-ablation experiment below confirms that the gap is occupational composition, not the anchor: with the same occupations held fixed and the Big Five anchor removed, Openness shifts by only *d* = −0.09, against the observational *d* = 2.09 in Table 8, and its ICC rises from 0.845 to 0.895 (Table 9 and Table 10).

RIASEC ICC drops were more modest (e.g., Realistic from 0.977 to 0.874; Enterprising from 0.955 to 0.606), leaving reliability in the good-to-excellent range even without personality anchors. The strata differ systematically in occupational content, so these between-stratum differences cannot be attributed to anchor availability alone; occupational composition also varies across strata. Even so, the covariation is several times larger on Big Five than on RIASEC, pointing to a stronger calibration role for the personality anchors than for the interest anchors.

To separate anchor availability from occupational content, we re-scored the 200-occupation subsample under the three masked anchor conditions (Table 9 and Table 10). RIASEC reliability was robust to anchor removal: ICC(2,1) stayed in the good-to-excellent range on all six dimensions, even with no anchor (0.735 to 0.918). Removing the Big Five anchors did not lower Big Five reliability, which was unchanged or higher than under the full-anchor baseline (e.g., Agreeableness 0.661 to 0.834). The score shifts were concentrated on Conscientiousness and Neuroticism: with the Big Five anchors removed, Conscientiousness rose toward the scale ceiling (Cohen’s *d* = 1.21 in the RIASEC-only condition and 1.66 with no anchor) and Neuroticism fell toward the floor (*d* = −1.30 in both), whereas the other dimensions shifted little (|*d*| ≤ 0.56 for Big Five and |*d*| ≤ 0.05 for RIASEC). Removing the RIASEC anchors also raised the arbitration rate from 21.0% to 61.0% (Big-Five-only) and 57.5% (no anchor), indicating greater between-rater dispersion on RIASEC in the absence of its anchor. Held constant across occupations, the large between-stratum Big Five differences in Table 8 are, therefore, driven mainly by occupational composition rather than anchor availability.

*Model systematic bias.* We estimated rater effects with repeated-measures ANOVAs treating rater model (three levels) as a within-occupation factor for each dimension (Supplementary Material S3). For the six RIASEC dimensions, generalized $\eta^{2}$ for the rater main effect was negligible throughout (max 0.017; |Hedges’ *g*| ≤ 0.31 for all pairwise contrasts). Big Five rater effects were larger and concentrated on Neuroticism ($\eta^{2}$ = 0.228), Conscientiousness ($\eta^{2}$ = 0.103), and Agreeableness ($\eta^{2}$ = 0.077). Marginal means localized these shifts to Kimi, which rated Neuroticism and Agreeableness higher and Conscientiousness lower than the other two models (largest |Hedges’ *g*| = 1.32 for Neuroticism vs. DeepSeek; full pairwise table in Supplementary Material S3.2). These shifts were only partially translational: the ICC(3,1) values from Section 3.2 (Agreeableness 0.763, Conscientiousness 0.611, and Neuroticism 0.540) indicate good rank-order agreement on Agreeableness but moderate-to-fair agreement on Conscientiousness and Neuroticism, so Kimi’s deviations combined a mean shift with residual rank-order disagreement.

*Arbitration.* The 309 arbitrated occupations had expert-mean RIASEC profiles closer to the dimensional center than the 1327 expert-mean occupations, and the arbitration verdicts pulled them further toward the center: arbitrated scores were lower for Realistic (Δ = −1.65) and higher for Enterprising (Δ = +1.29) and Social (Δ = +0.91); other shifts on RIASEC were smaller (max |Δ| = 0.64 on Artistic). Big Five arbitration shifts stayed within ±0.45 on the 1–5 scale, in proportion to the smaller Big Five score range. Arbitration is triggered by between-rater disagreement, which is elevated for occupations with comparable expert loadings across multiple Holland categories; the verdicts, therefore, regress ambiguous profiles toward each dimension’s midpoint.

*Cross-cultural reference check.* A final descriptive comparison tests whether the framework reproduces its reference anchors or integrates them with Chinese occupational content. LLM RIASEC scores were compared with O*NET U.S. scores on the 1539 occupations whose Top-1 O*NET match score reached at least 0.85. Chinese scores were systematically higher on Social (*d* = 0.84), Conventional (*d* = 0.93), and Investigative (*d* = 0.60); differences on the remaining three dimensions were small to negligible (|*d*| ≤ 0.25; complete table in Supplementary Material S4.3). These deviations emerged even though O*NET values were supplied as anchors in the prompt, indicating that the agents did not merely reproduce the anchor values.

## 4. Discussion

The framework’s psychometric evidence diverged between RIASEC and Big Five. RIASEC met conventional thresholds for reliability, MTMM block separation, and convergent validity against O*NET, with only weak support on structural validity. Big Five evidence stratified along a single axis: absolute agreement was uniformly high, but ICC was attenuated on Conscientiousness and Neuroticism, the two dimensions whose between-occupation variance is compressed under the ideal-worker rating frame.

### 4.1. RIASEC Evidence Across Four Classes

For RIASEC, the four classes of psychometric evidence converge. Inter-rater reliability was excellent across all six dimensions and exceeded Cicchetti’s (1994) single-rater threshold; MTMM block separation between monotrait-heteromethod and heterotrait-heteromethod correlations was large; known-group differentiation was substantial on the four theoretically anchored major-category contrasts (Section 3.2, Section 3.3 and Section 3.4). External convergent correlations against O*NET ranged from *r* = 0.84 to 0.96 across the six dimensions, with a pooled *r* = 0.95 (Section 3.5), placing the framework’s outputs in the range of within- and cross-form agreement reported for the O*NET Interest Profiler family (Rounds et al., 2010). These reliability and MTMM results index agreement among three LLM raters that share substantial pretraining data and receive identical prompts and anchors. Their convergence, therefore, reflects consistency under partial common-source dependence rather than full rater independence.

Two first-letter agreement rates are numerically close. The three expert agents agreed on the first Holland letter for 83.5% of the reliability-sample occupations (Section 3.2). The final profile matched the O*NET first Holland letter for 83.8% of the crosswalked occupations (Section 3.5). The two rates measure different things. The first is internal inter-rater consistency among the three LLM agents. The second is an external cross-system match, also shaped by real cross-cultural differences between Chinese and U.S. occupational codings and by crosswalk noise. We, therefore, do not read the near-equality of the percentages as decomposing the 16.2% external non-match into internal and benchmark components. The two rates co-occur at a similar value; they are not equivalent.

The RIASEC results also cannot be read as a product of simple anchor copying. The known-group ANOVAs are organized around the 2022 Chinese occupational categories, not the U.S. occupational structure underlying the anchors; the predicted category-by-dimension pattern is nonetheless recovered on the four theoretically anchored RIASEC dimensions (Section 3.4). Had the agents reproduced anchor vectors, the known-group signal would have carried the U.S. occupational structure, not the Chinese taxonomy. A more direct piece of anchor-versus-judgment evidence, the systematically higher Chinese S and C scores than the matched O*NET values, is taken up in Section 4.3.

### 4.2. The Source of the Lower Big Five ICC

Big Five ICC(2,1) values for Conscientiousness and Neuroticism fell well below any RIASEC value. Read without context, these numbers might suggest that the three LLMs disagreed on Big Five scoring. They did not.

Three lines of evidence indicate that the low Big Five ICCs reflect the joint effect of variance compression and rater-level calibration differences, not large-scale rank-order disagreement among the three agents. First, the mean absolute deviations on Conscientiousness and Neuroticism were among the smallest in the entire 11-dimension set (Section 3.2). In absolute terms, Conscientiousness had the lowest MAD of any dimension. Neuroticism sat among the lowest alongside Realistic. Second, the within-occupation disagreement on these two dimensions was comparable to, or exceeded, the between-occupation variance (Section 3.2). The limiting factor is between-occupation variance, not within-occupation disagreement. This pattern fits the ideal-worker rating frame adopted in Section 2.2. For Neuroticism, however, the link is partly procedural rather than emergent. The scoring prompt explicitly instructed raters that, under the ideal-worker frame, most occupations should fall in a low Neuroticism range (1.5 to 2.5 on the 1–5 scale; Supplementary Material S1.2), so the restricted between-occupation variance on Neuroticism is at least partly built into the rating instruction rather than arising spontaneously from the conceptual frame. For Conscientiousness, no numeric range was prescribed, so its compression is more plausibly attributable to the frame itself. In neither case can the contributions of instruction and frame be separated with a single-frame design. Even so, the data establish the variance structure: when between-target variance is small, the ICC formula attenuates even when absolute agreement is high (Koo & Li, 2016). Third, switching from ICC(2,1) to ICC(3,1) raised Neuroticism from 0.374 to 0.540, Agreeableness from 0.678 to 0.763, and Conscientiousness from 0.522 to 0.611 (Section 3.2). The residual shortfall on Neuroticism after rater main effects are removed indicates some rank-order divergence, but it is modest relative to the two structural sources.

The source of the calibration offset is localized. Kimi-K2.5 rated Neuroticism higher, Agreeableness higher, and Conscientiousness lower than the other two agents. The largest shift occurred on Neuroticism (|Hedges’ *g*| ≈ 1.32; Section 3.6).

### 4.3. Anchor Effects and Their Limits

The anchor-ablation experiment clarifies what the Big Five anchors do. Holding occupations constant, removing the Big Five anchors did not lower Big Five reliability, so the lower ICC in the observational RIASEC-only stratum reflects that stratum’s occupational composition, not the missing anchor. What the anchors mainly do is set the level of the Conscientiousness and Neuroticism means. Without them, Conscientiousness drifts toward the ceiling and Neuroticism toward the floor, as expected, since the ideal-worker frame pushes almost every occupation toward high Conscientiousness and low Neuroticism. Openness, Extraversion, and Agreeableness, which vary more across occupations, change little. A likely reason the Big Five depend more on the anchors is that everyday text describing occupations says more about tasks and tools than about personality, so the models hold weaker priors for the Big Five and rely more on the reference values. The practical implication is unchanged: Conscientiousness and Neuroticism should be read as broad bands rather than fine rankings, and with attention to anchor coverage.

The anchors did not dictate the final scores. With the RIASEC anchors removed, RIASEC reliability stayed in the good-to-excellent range, and the scores shifted only moderately, so the agents reconstruct the RIASEC profile from the Chinese occupational content rather than inherit it from the anchors. The systematically higher Chinese scores on Social and Conventional than on the matched O*NET values (Section 3.6) are consistent with the agents integrating the anchors with the Chinese occupational definitions rather than reproducing them. Alternative mechanisms could, in principle, contribute to the same directional shift. A Chinese-language training corpus bias toward service- and regulation-oriented occupational language is one; a prompt-level effect of the ideal-worker frame on S and C specifically is another. Our data cannot decompose the observed offsets into these components. These data rule out one alternative: that the agents’ outputs are direct copies of the O*NET anchors on the dimensions covered by them.

### 4.4. Structural Validity

We evaluated structural validity with three pieces of evidence that bear on different properties: the hexagonal-order test of the circular RIASEC model, the known-group ANOVAs, and a cluster-based check against the administrative taxonomy. For RIASEC, the correspondence index was C = 0.653, below the empirical U.S. benchmark of approximately 0.70 reported for Holland’s circular order model in the meta-analytic evaluation by Tracey and Rounds (1993). The associated randomization p value was 0.136 (Section 3.4), failing to reject the null of random order at conventional significance. The ordinal gradient reported alongside the C value is descriptively consistent with a circular arrangement: adjacent-pair correlations exceed alternating-pair correlations, which in turn exceed opposite-pair correlations. That gradient cannot carry inferential weight against the nonsignificant test. One contributing factor to the sub-threshold C is the composition of the Chinese taxonomy: Realistic occupations account for more than half of the 1636 entries (Section 3.1), and when one type dominates, the inter-dimension correlations feeding into the C-index are reshaped, yielding different C values across differently composed samples even when the underlying structure is the same (Rounds & Tracey, 1996). Cross-cultural evidence on the hexagonal model has accordingly been mixed, with several non-U.S. samples falling below the U.S. benchmark; Long et al. (2005) reported partial support for the hexagonal model in Chinese university students, and our data echo this pattern at the occupational level.

A second, independent check, not tied to the hexagonal metric, comes from the cluster analysis (Section 3.4; full procedure and cluster profiles in Supplementary Material S3.1). The 11-dimensional RIASEC and Big Five profile space yielded a five-cluster solution whose agreement with the administrative taxonomy reached an adjusted Rand index of 0.418, well above the zero value expected under random partition. The test does not assume a hexagonal metric, so it is not subject to the same composition-driven attenuation that affected the C-index. The major-category labels were not part of the prompt, so the alignment does not arise from explicit label reuse; the agents did, however, receive occupation names and task descriptions that carry implicit category cues, so the ARI is best read as evidence that the psychological profile space respects the structure of the major categories suggested by those descriptions rather than as an independence test. The randomization test is the only direct test of the circular model, and it was nonsignificant. The known-group ANOVAs and the cluster analysis bear on different properties: whether the dimensions differentiate occupational categories as theory predicts, and whether the profile space recovers the administrative taxonomy. These establish that the profiles are meaningfully structured and theory-consistent, but they are not tests of the hexagonal order and do not substitute for it.

Big Five occupation-level intercorrelations matched the expected sign pattern on two of the four reference contrasts. Neuroticism correlated negatively with Conscientiousness, and Extraversion correlated positively with Agreeableness, both in the direction predicted by meta-analytic expectations (van der Linden et al., 2010). The Neuroticism–Agreeableness correlation and the Agreeableness–Conscientiousness correlation reversed sign relative to individual-level expectations (Section 3.4). We read this primarily as a level-of-analysis effect: aggregation over incumbents shifts what the correlation indexes from within-person trait covariance to between-occupation covariance among the trait profiles required by jobs (Klein & Kozlowski, 2000). A second contributor may be range restriction on Conscientiousness and Neuroticism (Section 3.2): sharply restricted variance leaves the residual covariation dominated by the few occupations that deviate from the near-ceiling or near-floor mass, and sign reversals relative to the unrestricted distribution become possible. Both reversed contrasts involve at least one restricted-range dimension, which is consistent with this mechanism. Distinguishing the two accounts would require pairing occupation-level scores with individual-level data; we leave this for future work.

### 4.5. Arbitration and Score Centering

Do the arbitrator verdicts represent productive averaging of marginal cases, or a source of conservative bias that compresses the score distribution? The data allow a partial answer in each direction.

Two pieces of evidence point toward productive averaging rather than arbitrator-induced bias. First, the arbitrator did not reverse the direction of the pre-arbitration expert signal. The expert mean already placed arbitrated occupations closer to the dimensional center than non-arbitrated occupations, and the arbitrator’s verdict preserved that rank order (Section 3.6). The verdicts, therefore, continue the experts’ collective reading on ambiguous occupations rather than overriding it. Second, arbitration is triggered by between-rater disagreement, and such disagreement concentrates on occupations that straddle adjacent Holland categories, for example, Realistic–Investigative or Social–Enterprising. Holland’s theory characterizes boundary occupations as mixed-type rather than single-type, with profiles blended across the two adjacent types rather than concentrated on one of them (Nauta, 2010). Final scores closer to the dimensional center for such occupations are consistent with Holland’s account of their hexagonal position, not a by-product of the arbitration rule.

Arbitration also reduces between-occupation variance among the arbitrated cases. The magnitude of this centering effect depends on the σ thresholds (*σ* > 0.6 on RIASEC and *σ* > 0.5 on Big Five) and the resulting 18.9% arbitration rate, which were set by design convention rather than empirical optimization. The centering we observed in the arbitrated subset fits averaging of boundary profiles more than broad conservative bias.

### 4.6. Practical Implications

The framework’s outputs have several applications in the Chinese occupational context. Person–job matching can use the RIASEC six-vector through standard Holland congruence indices (Brown & Gore, 1994); the two Holland high-point codes reported for each occupation match the formats used in standard Holland-based counseling (Nauta, 2010). Workforce research and labor-market analysis can use the profiles to characterize Chinese occupational segments that have not previously been described at the occupation level. For Big Five applications, absolute agreement is uniformly high, and external convergent validity is adequate for Openness and Extraversion (Section 3.5). Applications that rely on fine-grained rank ordering of occupations on Conscientiousness or Neuroticism should treat the scores as differentiating broad occupational bands rather than supporting narrow within-band rank ordering, given the variance compression documented in Section 3.2 and Section 4.2.

### 4.7. Limitations and Future Directions

Several limitations of the present study suggest corresponding extensions.

First, the three expert agents differ in both model identity and temperature parameter. This is a deliberate trade-off: by varying both factors simultaneously, we maximize rater heterogeneity and mirror the ways human raters differ in applied settings, but we forfeit the ability to decompose residual variance into a model-identity component and a temperature component. A factorial design that orthogonally varies the two factors would make this decomposition possible and is a natural methodological follow-up.

Second, no independent human ratings or incumbent data were collected for the Chinese occupations themselves. All convergent evidence rests on crosswalked foreign benchmarks (O*NET, Anni et al., 2025). High agreement among the three agents, therefore, establishes inter-rater consistency, and agreement with these benchmarks establishes cross-system convergence, but neither establishes correctness against Chinese occupational reality in the absence of a domestic criterion. The results are accordingly best read as agreement with external references rather than as validation against ground truth. The next concrete step is a domestic human-rating study: a stratified subset of the 1636 occupations (sampled across major categories and arbitration status) rated by trained Chinese vocational-psychology experts, providing an internal convergent anchor. Such a study would also disambiguate the weak Agreeableness coefficient (*r* = 0.245 against Anni et al., 2025; Section 3.5), since Agreeableness is the most culturally variable of the Big Five (Schmitt et al., 2007) and the crosswalk-based design cannot separate cross-cultural noise from a framework limitation.

Third, the framework was evaluated on a single occupational taxonomy in a single national and linguistic context. Generalization to other classifications (for example, the U.S. O*NET-SOC 2019 or the ISCO-08 catalogue) would test the generality of the reliability and validity results.

Fourth, the results are bound to the specific LLMs used at the time of scoring. The framework itself is model-agnostic, but the empirical findings may shift when the underlying models are updated. Longitudinal rescoring with newer model releases would test the temporal stability of the profiles.

Fifth, the rank-ordering limitation on Conscientiousness and Neuroticism is consistent with the ideal-worker rating frame restricting between-occupation variance on these dimensions, but a single-frame design cannot establish causation. A head-to-head comparison of the ideal-worker frame, a typical-worker frame, and a hybrid frame on the same target occupations would adjudicate this and clarify how framing choices propagate into the variance structure.

Sixth, the architecture can be extended to other occupational attributes such as skills, knowledge requirements, and work values, where similar variance compression and anchoring issues may arise.

Seventh, the arbitration thresholds (*σ* > 0.6 for RIASEC, *σ* > 0.5 for Big Five) were set by convention, not tuned. Lower thresholds would send more occupations to arbitration and, because arbitration centers ambiguous profiles, compress between-occupation variance further and pull profiles toward the scale midpoints. Higher thresholds would do the reverse: more occupations stay at the expert mean, preserving dispersion but leaving more rater disagreement unresolved. A threshold sweep would quantify this trade-off and is a useful robustness check for future work.

### 4.8. Conclusions

The multi-agent LLM framework can produce large-scale occupational psychological profiles whose psychometric standing differs by construct family. For RIASEC, internal indices meet conventional reliability thresholds, and external convergent correlations with O*NET place the framework’s outputs in the same range as the U.S. human benchmark; the strict circular structure received only weak support, though the dimensions differentiated occupational categories, and the profile space recovered the administrative taxonomy. For the Big Five, absolute agreement is uniformly high; ICC is lower on the dimensions whose between-occupation variance is restricted in our sample, and reliability is sensitive to the availability of the personality anchors during scoring.

Three methodological takeaways follow for future LLM-as-rater work.

First, reliability should be evaluated with a multi-index strategy. ICC alone can misrepresent rater agreement under restricted between-target variance, attenuating even when absolute agreement is high (McGraw & Wong, 1996; Koo & Li, 2016). Reporting an absolute-agreement index alongside ICC, and a consistency coefficient such as ICC(3,1) when model-level calibration offsets are suspected, separates rater disagreement from variance restriction and rater main effects.

Second, when reference data are available, they can function as calibration anchors that align raters’ cognitive categorization of the rating dimensions while raters retain authority over the final score. This is an in-context analog of frame-of-reference training in the human-rater literature, where standardized exemplars align rater judgments without dictating any single one (Sulsky & Day, 1992; Lievens, 2001).

Third, rating-frame choices, such as the ideal-worker versus typical-worker distinction adopted here, should be treated as active design decisions. Their direction is theoretically grounded, but their magnitude requires head-to-head comparison.

Alongside these methodological contributions, the framework produces a public database of RIASEC and Big Five profiles for all 1636 occupations in the 2022 Chinese Occupational Classification. To our knowledge, this is the first occupation-level psychological profile set for the Chinese taxonomy. It is ready for use in person–job matching, career counseling, and Chinese labor-market research.

## Figures and Tables

**Table 1:** RIASEC descriptive statistics for 1636 Chinese occupations.

Dimension	*M*	*SD*	Min	Max	Skewness	Kurtosis	*Mdn*
Realistic (R)	5.37	1.85	1.0	7.0	−1.01	−0.40	6.303
Investigative (I)	3.77	1.55	1.0	7.0	0.38	−1.11	3.43
Artistic (A)	2.07	1.41	1.0	7.0	2.00	3.41	1.505
Social (S)	2.65	1.49	1.0	7.0	1.26	0.62	2.07
Enterprising (E)	2.36	1.39	1.0	7.0	1.51	1.53	1.83
Conventional (C)	4.89	0.77	2.0	7.0	0.06	0.03	4.83

*Note. M* = mean; *SD* = standard deviation; *Mdn* = median. Scores are on a 1–7 Likert scale. *n* = 1636 occupations from the 2022 Chinese Occupational Classification.

**Table 2:** Big Five descriptive statistics for 1636 Chinese occupations.

Dimension	*M*	*SD*	Min	Max	Skewness	Kurtosis	*Mdn*
Openness (O)	3.31	0.65	2.0	4.7	−0.07	−1.11	3.404
Conscientiousness (C)	4.43	0.21	3.4	4.9	−0.59	1.202	4.43
Extraversion (E)	2.74	0.56	1.7	4.7	0.57	−0.14	2.707
Agreeableness (A)	3.43	0.43	2.5	4.7	0.505	−0.16	3.404
Neuroticism (N)	1.85	0.19	1.3	3.2	0.73	4.51	1.87

*Note.* Scores are on a 1–5 Likert scale rescaled from Anni et al. (2025) via Zhang et al. (2022) Chinese employee-sample norms; see Section 2.1 and Supplementary Material S4. Statistics are based on all 1636 occupations of the 2022 Chinese Occupational Classification.

**Table 3:** Inter-rater reliability across three LLM raters: ICC, 95% confidence Intervals, and mean absolute deviation by dimension.

Dimension	*N*	ICC(2,1) [95% CI]	ICC(2,*k*) [95% CI]	ICC(3,1) [95% CI]	MAD (% Scale)
RIASEC-R	1626	0.979 [0.98, 0.98]	0.993 [0.99, 0.99]	0.979 [0.98, 0.98]	0.137 (2.3%)
RIASEC-I	1626	0.927 [0.85, 0.96]	0.974 [0.94, 0.99]	0.951 [0.95, 0.96]	0.257 (4.3%)
RIASEC-A	1626	0.948 [0.89, 0.97]	0.982 [0.96, 0.99]	0.964 [0.96, 0.97]	0.186 (3.1%)
RIASEC-S	1626	0.937 [0.85, 0.97]	0.978 [0.94, 0.99]	0.961 [0.96, 0.96]	0.237 (4.0%)
RIASEC-E	1626	0.956 [0.94, 0.97]	0.985 [0.98, 0.99]	0.963 [0.96, 0.97]	0.181 (3.0%)
RIASEC-C	1626	0.866 [0.85, 0.88]	0.951 [0.95, 0.96]	0.869 [0.86, 0.88]	0.179 (3.0%)
BIG FIVE-O	1626	0.871 [0.86, 0.88]	0.953 [0.95, 0.96]	0.873 [0.86, 0.88]	0.156 (3.9%)
BIG FIVE-C	1626	0.522 [0.34, 0.65]	0.766 [0.61, 0.85]	0.611 [0.59, 0.64]	0.115 (2.9%)
BIG FIVE-E	1626	0.842 [0.79, 0.88]	0.941 [0.92, 0.95]	0.862 [0.85, 0.87]	0.152 (3.8%)
BIG FIVE-A	1626	0.678 [0.48, 0.79]	0.864 [0.73, 0.92]	0.763 [0.75, 0.78]	0.182 (4.6%)
BIG FIVE-N	1626	0.374 [0.12, 0.56]	0.642 [0.29, 0.79]	0.540 [0.51, 0.57]	0.142 (3.6%)

*Note.* Three raters = DeepSeek-V3.2, GLM-5.1, Kimi-K2.5. *n* = 1626 occupations with complete triplets. ICC(2,1) and ICC(2,*k*) = two-way random-effects absolute-agreement coefficients. ICC(3,1) = two-way mixed-effects consistency coefficient (removes rater main effects). MAD = mean absolute deviation across raters, reported in raw units and as a percentage of scale range (1–7 for RIASEC, 1–5 for Big Five).

**Table 4:** Known-group predictions: major-category-by-dimension mean, rank, and theoretical match.

Major Category	Dimension	*M*	Rank Within Dimension	Match
Professional and Technical	Investigative	5.36	1	✓
Managerial	Enterprising	6.44	1	✓
Managerial	Social	5.68	1	✓
Manufacturing and Related	Realistic	6.74	1	✓
Agriculture and Forestry	Realistic	6.61	2	✓
Clerical and Related	Conventional	5.62	1	✓

*Note.* Predictions are derived from Holland’s (1997) theory of vocational personalities, which maps each RIASEC dimension to specific occupational environments. Each prediction specifies that the named industry group should score highest (rank = 1) or near-highest (rank = 2) on the named dimension. ✓ indicates the predicted ranking was observed. All six a priori predictions were confirmed. The six predictions cover five major occupational categories; the Managerial category enters two predictions (Enterprising and Social).

**Table 5:** Big Five occupation-level factor intercorrelation matrix.

	O	C	E	A	N
O	—	−0.50	0.63	0.53	0.65
C	−0.50	—	−0.43	−0.43	−0.76
E	0.63	−0.43	—	0.73	0.505
A	0.53	−0.43	0.73	—	0.48
N	0.65	−0.76	0.505	0.48	—

*Note.* O = Openness; C = Conscientiousness; E = Extraversion; A = Agreeableness; N = Neuroticism. Pearson correlations across 1636 occupation-level profiles. All |r| ≥ 0.43 exceed p < 0.001.

**Table 6:** RIASEC convergent validity against O*NET Interest Profiler.

Dimension	*N*	*r*	95% CI	Mean Difference (LLM − O*NET)
Realistic	1552	0.956	[0.952, 0.960]	0.07
Investigative	1552	0.886	[0.875, 0.896]	0.43
Artistic	1552	0.9200	[0.912, 0.927]	0.14
Social	1552	0.932	[0.925, 0.938]	0.44
Enterprising	1552	0.922	[0.914, 0.929]	−0.03
Conventional	1552	0.843	[0.828, 0.857]	0.42

*Note. n* = 1552 Chinese occupations matched to at least one O*NET-SOC 2019 entry via the Section 2.1 crosswalk. Pearson r between LLM-generated RIASEC scores and O*NET Interest Profiler scores. 95% CIs computed via Fisher *r*-to-*z* transformation. All correlations significant at *p* < 0.001. Mean difference is on the 1–7 RIASEC scale.

**Table 7:** Big Five convergent validity against Anni et al. (2025).

Dimension	*N*	*r*	95% CI	Mean Difference (LLM − Anni et al.)
Openness	798	0.740	[0.707, 0.770]	0.07
Conscientiousness	798	0.372	[0.311, 0.430]	0.69
Extraversion	798	0.640	[0.597, 0.679]	−0.17
Agreeableness	798	0.245	[0.179, 0.309]	−0.19
Neuroticism	798	0.344	[0.281, 0.404]	−0.79

*Note.* n = 798 Chinese occupations matched to at least one Anni et al. (2025) ISCO-08 entry via the Section 2.1 crosswalk. Pearson r between LLM-generated Big Five scores and the rescaled Anni et al. Big Five scores (rescaling described in Section 2.1 and Supplementary Material S4.1). 95% CIs computed via Fisher *r*-to-*z* transformation. All correlations significant at *p* < 0.001. Mean difference is on the 1–5 BFI-2 metric.

**Table 8:** Sensitivity of scores to anchor-coverage stratum: full-anchor vs. RIASEC-only comparison on all 11 dimensions.

Dimension	Full-Anchor *M* (*SD*)	RIASEC-Only *M* (*SD*)	Cohen’s *d*	*t*	Δ*M*
BIG FIVE-O	3.50 (0.55)	2.53 (0.35)	2.09	29.79	0.97
BIG FIVE-C	4.39 (0.19)	4.64 (0.13)	−1.54	−21.95	−0.25
BIG FIVE-E	2.90 (0.50)	2.08 (0.21)	2.11	28.25	0.81
BIG FIVE-A	3.55 (0.38)	2.92 (0.19)	2.11	28.96	0.63
BIG FIVE-N	1.90 (0.17)	1.63 (0.09)	1.97	27.47	0.27
RIASEC-R	5.06 (1.92)	6.72 (0.39)	−1.20	−15.38	−1.66
RIASEC-I	4.01 (1.58)	2.74 (0.78)	1.03	14.00	1.28
RIASEC-A	2.25 (1.50)	1.34 (0.48)	0.81	10.64	0.91
RIASEC-S	2.91 (1.53)	1.52 (0.41)	1.25	16.12	1.39
RIASEC-E	2.59 (1.45)	1.38 (0.28)	1.16	14.87	1.21
RIASEC-C	4.90 (0.82)	4.81 (0.48)	0.14	1.96	0.09

*Note.* Full-anchor n = 1310 occupations that received both RIASEC and Big Five reference values; RIASEC-only n = 318 occupations that received RIASEC anchors only. Independent-samples *t*-tests with Cohen’s d. All *t*-tests significant at *p* < 0.001 except RIASEC-C (*p* = 0.050). ΔM = mean difference (full-anchor − RIASEC-only) on the original scale (1–7 for RIASEC, 1–5 for Big Five). Between-stratum differences conflate anchor availability with occupational content, and the large |*d*| values are further inflated by the restricted within-stratum *SD* of the RIASEC-only group; Δ*M* is the more interpretable index.

**Table 9:** Anchor-ablation experiment: inter-rater reliability ICC(2,1) under four anchor conditions.

Dimension	Full	RIASEC-Only	Big-Five-Only	No Anchor
RIASEC-R	0.972	0.973	0.910	0.918
RIASEC-I	0.926	0.924	0.867	0.875
RIASEC-A	0.942	0.935	0.891	0.901
RIASEC-S	0.941	0.950	0.903	0.918
RIASEC-E	0.954	0.960	0.890	0.898
RIASEC-C	0.860	0.880	0.706	0.735
Big Five-O	0.845	0.895	0.835	0.892
Big Five-C	0.409	0.569	0.246	0.460
Big Five-E	0.834	0.875	0.822	0.853
Big Five-A	0.661	0.834	0.707	0.787
Big Five-N	0.239	0.248	0.182	0.298

*Note. n* = 200 occupations drawn from the full-anchor stratum. ICC(2,1) = two-way random-effects, absolute-agreement, single-rater intraclass correlation across the three LLM raters, recomputed within each anchor condition. Full = both anchors injected (main-pipeline scores); RIASEC-only = Big Five anchors masked; Big-Five-only = RIASEC anchors masked; No anchor = both anchors masked.

**Table 10:** Anchor-ablation experiment: score shifts from the full-anchor baseline under three masked conditions.

Dimension	Full *M* (*SD*)	Masked *M* (*SD*)	Δ*M*	*t*	*d*
***RIASEC-only***					
RIASEC-R	5.20 (1.81)	5.18 (1.82)	−0.01	−0.78	−0.01
RIASEC-I	4.04 (1.61)	4.04 (1.59)	+0.00	+0.03	+0.00
RIASEC-A	2.18 (1.37)	2.20 (1.38)	+0.02	+1.27	+0.01
RIASEC-S	2.94 (1.64)	2.91 (1.66)	−0.03	−1.99 *	−0.02
RIASEC-E	2.48 (1.38)	2.48 (1.40)	+0.00	+0.11	+0.00
RIASEC-C	4.86 (0.78)	4.83 (0.77)	−0.04	−2.58 *	−0.05
Big Five-O	3.50 (0.54)	3.44 (0.76)	−0.06	−3.00 **	−0.09
Big Five-C	4.39 (0.18)	4.60 (0.17)	+0.21	+23.03 ***	+1.21
Big Five-E	2.85 (0.50)	2.71 (0.64)	−0.15	−9.78 ***	−0.26
Big Five-A	3.56 (0.39)	3.29 (0.55)	−0.27	−15.92 ***	−0.56
Big Five-N	1.89 (0.14)	1.71 (0.12)	−0.17	−22.17 ***	−1.30
***Big-Five-only***					
RIASEC-R	5.20 (1.81)	4.78 (1.81)	−0.42	−9.69 ***	−0.23
RIASEC-I	4.04 (1.61)	4.68 (1.47)	+0.64	+17.12 ***	+0.41
RIASEC-A	2.18 (1.37)	2.76 (1.41)	+0.58	+18.47 ***	+0.41
RIASEC-S	2.94 (1.64)	3.59 (1.53)	+0.65	+18.89 ***	+0.41
RIASEC-E	2.48 (1.38)	2.79 (1.23)	+0.31	+9.01 ***	+0.24
RIASEC-C	4.86 (0.78)	5.26 (0.70)	+0.39	+11.55 ***	+0.53
Big Five-O	3.50 (0.54)	3.53 (0.57)	+0.03	+2.92 **	+0.06
Big Five-C	4.39 (0.18)	4.36 (0.18)	−0.04	−4.08 ***	−0.20
Big Five-E	2.85 (0.50)	2.92 (0.49)	+0.07	+7.31 ***	+0.15
Big Five-A	3.56 (0.39)	3.61 (0.41)	+0.05	+4.23 ***	+0.12
Big Five-N	1.89 (0.14)	1.93 (0.15)	+0.04	+4.77 ***	+0.27
***No anchor***					
RIASEC-R	5.20 (1.81)	4.81 (1.83)	−0.39	−8.74 ***	−0.21
RIASEC-I	4.04 (1.61)	4.76 (1.52)	+0.72	+17.38 ***	+0.46
RIASEC-A	2.18 (1.37)	2.75 (1.44)	+0.57	+17.45 ***	+0.40
RIASEC-S	2.94 (1.64)	3.53 (1.56)	+0.59	+16.70 ***	+0.37
RIASEC-E	2.48 (1.38)	2.75 (1.30)	+0.27	+8.14 ***	+0.20
RIASEC-C	4.86 (0.78)	5.37 (0.71)	+0.50	+13.68 ***	+0.67
Big Five-O	3.50 (0.54)	3.53 (0.78)	+0.03	+1.36	+0.04
Big Five-C	4.39 (0.18)	4.66 (0.15)	+0.27	+28.26 ***	+1.66
Big Five-E	2.85 (0.50)	2.78 (0.66)	−0.07	−4.20 ***	−0.12
Big Five-A	3.56 (0.39)	3.36 (0.55)	−0.20	−11.28 ***	−0.42
Big Five-N	1.89 (0.14)	1.70 (0.15)	−0.19	−21.02 ***	−1.30

*Note. n* = 200 occupations drawn from the full-anchor stratum, re-scored under three masked anchor conditions: RIASEC-only = Big Five anchors masked; Big-Five-only = RIASEC anchors masked; no anchor = both anchors masked. Full = both anchors injected (main-pipeline scores). *M* and *SD* are on the original scale (1–7 for RIASEC, 1–5 for Big Five). Δ*M* = masked − full; positive values indicate a higher score than under full anchors. *t* is the paired-samples *t* (*df* = 199); *d* is Cohen’s *d* for the same contrast. * *p* < 0.05, ** *p* < 0.01, *** *p* < 0.001.

## Data Availability

All prompts, R and Python code, raw outputs, and the per-occupation RIASEC and Big Five dataset for the 1636 occupations are openly available at https://osf.io/gdjb4/ (accessed on 21 June 2026).

## Supplementary Materials

The following supporting information can be downloaded at: https://www.mdpi.com/article/10.3390/bs16071064/s1. Supplementary Material S1: Full Prompt Specifications; Supplementary Material S2: End-to-End Worked Example; Supplementary Material S3: Cluster and Rater-Bias Analyses; Supplementary Material S4: Supporting Data for Big Five Rescaling, MTMM, and Cross-Cultural Comparison. References (Bakeman, 2005) and (Olejnik & Algina, 2003) are citied in the Supplementary Materials.
