# Supplementary material for: Multi-Agent LLMs for Occupational Profiling: Psychometric Validation on 1636 Chinese Occupations
Source: Behav Sci (Basel). 2026 Jun 26;16(7):1064. doi: 10.3390/bs16071064 (PMC13405925; doi:10.3390/bs16071064)
Supplement: Supplementary file 1 [file behavsci-16-01064-s001.zip › Supplementary_Material_S2.pdf]

Supplementary Material S2: End-to-End Worked Example

This supplement documents the node-by-node trace of the multi-agent pipeline on two Chinese occupations drawn from the 2022 *Classification of Occupations of the People's Republic of China*. Case 1 (*Psychological Counselor*, 2-07-10-03) runs through the default path: the three expert agents converge closely enough that Node 5 does not flag the occupation for arbitration, and the final profile is the three-expert mean. Case 2 (*Director*, 2-09-01-06) runs through the arbitration path. Node 5 flags two RIASEC dimensions on which the three experts disagree, and Node 6 invokes the arbitrator. The arbitrator independently re-renders scores for all 11 dimensions. Its verdicts track the three-expert means closely on the non-disputed dimensions and resolve the two disputed ones with reasoned adjudication. Both cases are traced here as produced by the pipeline; all numeric values and justifications are reproduced from the per-occupation JSON records rather than re-derived for presentation.

The structure of this supplement mirrors the seven-node workflow in Figure 1 of the main text. For each case we report the Node 1 input, the Node 2 matcher output, the Node 3 aggregated anchor, the Node 4 three-expert ratings, the Node 5 statistics check, the Node 6 arbitration verdict with rationale (for Case 2 only), and the Node 7 final profile. Each section ends with a brief annotation describing what the case contributes to the interpretive arguments made in Sections 4.2, 4.3, and 4.5 of the main text.

Case 1. Psychological Counselor (心理咨询师, 2-07-10-03), Non-Arbitrated Path

Input (Node 1 stage: raw occupation record)

Occupation code: 2-07-10-03

Occupation name: 心理咨询师 (Psychological Counselor)

Definition (translated): A professional who applies psychological counseling techniques and methods to analyze, assess, counsel, and help resolve the psychological problems of clients.

Principal tasks (translated, five items):

1. Conducting interviews with clients to understand their psychological state.
2. Analyzing information provided by the client to identify causes and cognitive factors behind the psychological problem.

3. Determining the entry point and method for counseling, and guiding the client to resolve the problem.
4. Using psychometric tests to assess the client and to interpret test results.
5. Proposing problem-resolution approaches tailored to the client's personality.

Node 1: Retrieval

The occupation title is embedded with Qwen3-Embedding-8B and queried against the persistent ChromaDB index of all O*NET-SOC 2019 occupations (cosine, HNSW). The top 50 candidates are retrieved and re-ranked against the full query text (name + definition + tasks) with Qwen3-Reranker-8B; the top 20 are passed downstream to Node 2.

Node 2: Agent 0 Matcher (DeepSeek-V3, T = 0.1)

Agent 0 selects the top five candidates. For each candidate Agent 0 returns a match score in [0, 1] together with a per-dimension justification covering work content, skill requirements, knowledge domain, work environment, and occupational level.

Table S2.1

*Case 1, Top-five matched O*NET occupations.*

Rank	O*NET-SOC code	Title	Match score
1	21-1014.00	Mental Health Counselors	0.95
2	19-3033.00	Clinical and Counseling Psychologists	0.90
3	21-1013.00	Marriage and Family Therapists	0.80
4	21-1019.00	Counselors, All Other	0.75
5	29-1129.00	Therapists, All Other	0.70

Representative Agent 0 justification, rank 1: "The work content is highly similar: both the Chinese definition and the O*NET occupation involve interviewing clients, analyzing psychological problems, and providing solutions. The Chinese task 'interviewing clients to understand their psychological state' corresponds to the O*NET task 'counsel clients and patients, individually and in group sessions.' Skill requirements, knowledge domain, and environment are also closely aligned." The full reasoning for all five candidates is available in the per-occupation JSON record.

Node 3: Data Enrichment and Anchor Aggregation

For each of the five candidates, Node 3 attempts to retrieve the RIASEC six-vector from the O*NET Interest Profiler and, via the O*NET-SOC 2019 → ISCO-08 crosswalk, the rescaled Big Five five-vector. Coverage is asymmetric for this occupation. Three candidates (21-1014.00 *Mental Health Counselors*, 19-3033.00 *Clinical and Counseling Psychologists*, 21-1013.00 *Marriage and Family Therapists*) have RIASEC entries; the two All Other residual categories (21-1019.00 *Counselors, All Other*; 29-1129.00 *Therapists, All Other*) do not. All five candidates map to ISCO-08 codes with valid Big Five scores. Candidates with available data are aggregated through inverse-distance weighting on cosine vector distance. RIASEC and Big Five weight-normalizations run independently: the three RIASEC-bearing candidates share the RIASEC weight pool, and all five ISCO-mapped candidates share the Big Five weight pool. Neither aggregate is diluted by missing entries in the other.

Table S2.2

Case 1, Aggregated reference anchor payload.

Metric	RIASEC						Big Five				
	R	I	A	S	E	C	O	C	E	A	*N*
**RIASEC (1–7)**	1.28	5.12	3.23	6.53	2.46	3.28					
**Big Five (1–5)**							3.70	3.60	3.35	3.94	2.68

The anchor profile identifies Social (S = 6.53) and Investigative (I = 5.12) as the dominant dimensions, consistent with the Holland literature on counseling-type occupations. The Big Five anchor sits slightly above the 1–5 midpoint on Agreeableness (A = 3.94) and Openness (O = 3.70), and slightly below it on Neuroticism (N = 2.68).

Node 4: Three-Expert Parallel Scoring

The three expert agents, vocational psychologist (DeepSeek-V3.2, T = 0.3), HR specialist (GLM-5.1, T = 0.5), and career counselor (Kimi-K2.5, T = 0.7), receive the same occupation input and anchor payload. Each returns a clamped score on all 11 dimensions with a brief per-dimension justification.

Table S2.3

Case 1, Ratings returned by the three expert agents.

Dim.	Scale	Vocational psychologist (DeepSeek-V3.2, $T = 0.3$)	HR specialist (GLM-5.1, $T = 0.5$)	Career counselor (Kimi-K2.5, $T = 0.7$)	Mean	*SD*
**RIASEC-R**	1–7	1.5	1.2	1.2	1.30	0.14
**RIASEC-I**	1–7	5.8	5.5	5.5	5.60	0.14
**RIASEC-A**	1–7	3.5	3.5	3.5	3.50	0.00
**RIASEC-S**	1–7	6.8	7.0	6.8	6.87	0.09
**RIASEC-E**	1–7	2.2	2.5	2.8	2.50	0.24
**RIASEC-C**	1–7	3.8	3.0	3.2	3.33	0.34
**Big Five-O**	1–5	4.2	4.5	3.9	4.20	0.24
**Big Five-C**	1–5	4.0	4.2	3.9	4.03	0.12
**Big Five-E**	1–5	3.3	3.0	3.2	3.17	0.12
**Big Five-A**	1–5	4.5	4.5	4.1	4.37	0.19
**Big Five-N**	1–5	1.8	1.5	2.2	1.83	0.29

Note. SD is the population standard deviation across the three raters (computed with $N = 3$ in the denominator), consistent with Node 5's implementation.

Representative per-dimension justification, Social (vocational psychologist): "The foundation of this profession is forming a therapeutic alliance, deep empathy, and the provision of professional support." Representative Neuroticism justification (HR specialist): "A counselor must remain extremely emotionally stable to serve as a container for the client's negative affect; losing composure under pressure is disqualifying."

Node 5: Statistics Check

Node 5 computes dimensionwise means and population *SD*s (see table above) and checks for arbitration triggers. The arbitration trigger is any RIASEC $SD > 0.6$ or any Big Five $SD > 0.5$. In this case the largest RIASEC *SD* is 0.34 (Conventional) and the largest Big Five *SD* is 0.29 (Neuroticism). Both are well below their respective thresholds.

Outcome: needs_arbitration: false. All 11 dimensions are flagged with overall confidence high. Node 6 is skipped, and the pipeline proceeds directly to Node 7 with the expert mean as the source.

Node 7: Final Profile

Table S2.4

Case 1, Final profile.

	RIASEC						Big Five				
	**R**	**I**	**A**	**S**	**E**	**C**	**O**	**C**	**E**	**A**	**N**
**Final (expert mean)**	1.3	5.6	**3.5**	**6.9**	2.5	3.3	4.2	4.0	3.2	**4.4**	**1.8**
**Anchor (Node 3)**	1.28	5.12	3.23	6.53	2.46	3.28	3.70	3.60	3.35	3.94	2.68
**Δ (final − anchor)**	+0.02	+0.48	+0.27	+0.34	+0.04	+0.05	+0.50	+0.43	−0.18	+0.43	**−0.85**

Holland top-3 code (standard): SIA.

Proportional high-point code (dimensions above 0.17 × total): SI.

Source: expert_average.

Annotation: What Case 1 illustrates

The deltas in the bottom row of Table S2.4 demonstrate *calibration without replacement* (Section 4.3 of the main text) on a single occupation. The agents preserve the anchor's dominant-dimension structure: Social stays the top RIASEC dimension and Investigative the second. Magnitudes shift upward on Social, Investigative, Artistic, Agreeableness, and Openness, and downward on Neuroticism. The Neuroticism deviation is the largest in magnitude (−0.85). Its source is visible in the raters' justifications. Each of the three agents invokes the ideal-worker principle to push Neuroticism below the anchor, which reflects incumbent states including stress. The profile is anchored in the external reference but not dictated by it.

Case 2. Director (daoyan, 导演, 2-09-01-06), Arbitrated Path

Input (Node 1 stage: raw occupation record)

Occupation code: 2-09-01-06

Occupation name: 导演 (Director)

Definition (translated): A professional engaged in music conducting and in directing the artistic performance of film, theater, dance, *quyi*, acrobatics, singing, and instrumental arts.

Principal tasks (translated, five items):

Organizing creative staff to study and analyze the script; forming an overall artistic conception and master plan.

Guiding each department's creative work under a unified thematic and creative intent.

Coordinating with troupe, production, or film-crew heads to determine casting and production venue.

Organizing and directing rehearsals, performances, and shoots on schedule.

Supporting production-side promotion and marketing of the finished work.

Node 1 and Node 2: Retrieval and Matching

Same retrieval and matching pipeline as Case 1. Agent 0 returns the five candidates in Table S2.5.

Table S2.5

*Case 2, Top-five matched O*NET occupations.*

Rank	O*NET-SOC code	Title	Match score
1	27-2012.00	Producers and Directors	0.95
2	27-2012.04	Talent Directors	0.85
3	27-2041.00	Music Directors and Composers	0.70
4	27-2032.00	Choreographers	0.60

5	27-1011.00	Art Directors	0.50

The five candidates already span the theatrical, musical, choreographic, and visual directing sub-occupations, which is consistent with the broader scope of the Chinese *daoyan* title.

Node 3: Aggregated Anchor

Table S2.6

Case 2, Aggregated reference anchor payload.

Metric	RIASEC						Big Five				
	R	I	A	S	E	C	O	C	E	A	*N*
**RIASEC (1–7)**	2.37	2.17	**6.55**	3.44	**5.14**	3.12					
**Big Five (1–5)**							3.75	3.58	3.43	3.75	2.91

The anchor places the occupation at the Artistic × Enterprising apex (A = 6.55, E = 5.14). The remaining four dimensions (S = 3.44, C = 3.12, R = 2.37, I = 2.17) all sit below the dominant two. This apex position sets up the arbitration that follows. The three experts agree on Artistic and Enterprising but diverge on the lower-ranked Investigative (scripted analytic work) and Social (actor-direction) components. These two dimensions are where the Chinese daoyan taxonomy includes activities (script analysis, performer coaching) that not all of the English-language directing occupations emphasize.

Node 4: Three-Expert Parallel Scoring

Table S2.7

Case 2, Ratings returned by the three expert agents.

Dim.	Scale	Vocational psychologist (DeepSeek-V3.2, $T = 0.3$)	HR specialist (GLM-5.1, $T = 0.5$)	Career counselor (Kimi-K2.5, $T = 0.7$)	Mean	*SD*
**RIASEC-R**	1–7	2.0	2.0	2.5	2.17	0.24
**RIASEC-I**	1–7	4.0	2.5	2.5	3.00	0.71
**RIASEC-A**	1–7	7.0	7.0	6.5	6.83	0.24
**RIASEC-S**	1–7	5.0	3.5	3.5	4.00	0.71

**RIASEC-E**	1–7	6.0	6.0	5.5	5.83	0.24
**RIASEC-C**	1–7	3.5	3.0	3.0	3.17	0.24
**Big Five-O**	1–5	4.8	4.8	4.2	4.60	0.28
**Big Five-C**	1–5	4.0	4.2	4.0	4.07	0.09
**Big Five-E**	1–5	4.2	3.8	3.8	3.93	0.19
**Big Five-A**	1–5	3.0	3.0	3.5	3.17	0.24
**Big Five-N**	1–5	2.2	2.0	2.2	2.13	0.09

Bold rows mark the two RIASEC dimensions whose $SD > 0.6$, the arbitration threshold.

The disagreement has a substantive shape, not a stylistic one. On Investigative, the vocational psychologist reads “directing” as an intellectually analytic activity (studying the script, interpreting the role, structuring the artistic reading) and rates I = 4.0. The HR specialist and the career counselor read directing as an executional activity (rehearsing, coordinating, coaching performers) and rate I = 2.5. On Social, the same split recurs. The vocational psychologist reads director-to-actor communication as interpersonal facilitation and rates S = 5.0; the other two experts read it as non-service-oriented work-relationship management and rate S = 3.5. Each justification is internally coherent; the disagreement is about which face of a boundary occupation dominates, not about whether one rater is wrong.

Node 5: Statistics Check

RIASEC conflicts: **I** ($SD = 0.71$), **S** ($SD = 0.71$).

Big Five conflicts: none (maximum $SD = 0.28$ on Openness, below the threshold of 0.5).

Arbitration triggered: **yes.**

Node 5 passes the full state (occupation input, anchor payload, three full expert scores and justifications, and a summary of the disputed dimensions) to Node 6.

Node 6: Arbitration (DeepSeek-V3.2, $T = 0.2$)

The arbitrator receives the expert record. It is instructed to issue a final score on all 11 dimensions, not only the disputed ones, with a rationale that cites specific experts and anchor values. Supplementary Material S1 §S1.4.2 gives the full ARBITRATION_INSTRUCTION; the

paragraph below shows how the non-disputed dimensions are treated in practice. The disputed dimensions receive a reasoned adjudication.

Table S2.8

Case 2, Arbitration verdicts on the RIASEC dimensions with expert disagreement.

Dim.	Anchor	Pre-arb. mean	Arbitrator verdict	Arbitrator's adjudication (summary, translated from the JSON rationale)
**I**	2.17	3.00	**3.0**	"The vocational psychologist's reading (4.0) captures the real analytic depth of scripting and directorial interpretation, but overshoots the anchor range for directing occupations (I = 2.24 for *Producers and Directors* and I = 2.69 for *Art Directors* in the candidate set; the 2.17 value in the anchor footer is the inverse-distance-weighted aggregate across all five candidates). The HR specialist and career counselor (both 2.5) correctly distinguish this work from pure scientific research but underweight its analytic component. A verdict of 3.0 acknowledges the analytic work while preserving the distinction from research-type occupations."
**S**	3.44	4.00	**4.0**	"The psychologist's 5.0 reflects the genuine interpersonal facilitation involved in directing actors; the other two experts' 3.5 correctly notes that this is professional rather than service-oriented interaction. The anchor value (3.44) sits close to the HR/career counselor reading. However, the Talent Director sub-anchor (S = 4.27) and the Choreographer sub-anchor (S = 3.96), the two candidates most focused on performer direction, pull upward. A verdict of 4.0 acknowledges the substantive interpersonal component that the HR-style framing understates, without adopting the pure Social-type framing."

Arbitrator outputs for the non-disputed dimensions. Per the ARBITRATION_INSTRUCTION (see Supplementary Material S1 §S1.4.2), the arbitrator is required to return a final score for all 11 dimensions, not only those flagged as disputed. On the non-disputed dimensions the arbitrator independently re-rendered each score. The resulting values closely track the expert means but are not identical, because the arbitrator cites the candidate-anchor values and sometimes rounds toward the nearest anchor-consistent reading. RIASEC: R = 2.0 (pre-arb mean 2.17), A = 6.8 (6.83), E = 5.8 (5.83), C = 3.2 (3.17). Big Five: O = 4.6 (4.60), C = 4.1 (4.07), E = 3.9 (3.93), A = 3.2 (3.17), N = 2.1 (2.13). Any dimension the

arbitrator omitted or returned outside the valid range would be back-filled from the corresponding expert mean by the pipeline (implementation details in Supplementary Material S1 §S1.5.3). No back-fill was required for this occupation.

Node 7: Final Profile

Table S2.9

Case 2, Final profile.

	RIASEC						Big Five				
	**R**	**I**	**A**	**S**	**E**	**C**	**O**	**C**	**E**	**A**	**N**
**Final (expert mean)**	2.0	3.0	**6.8**	4.0	**5.8**	3.2	4.6	4.1	3.9	3.2	2.1
**Pre-arb. mean**	2.17	3.00	6.83	4.00	5.83	3.17	4.60	4.07	3.93	3.17	2.13
**Anchor (Node 3)**	2.37	2.17	6.55	3.44	5.14	3.12	3.75	3.58	3.43	3.75	2.91
**Δ (final − anchor)**	−0.37	+0.83	+0.25	+0.56	+0.66	+0.08	+0.85	+0.52	+0.47	−0.55	**−0.81**

Holland top-3 code (standard): AES.

Proportional high-point code: AE.

Source: arbitration.

Annotation: What Case 2 illustrates

Three features of this trace make it useful evidence for the methodological arguments in the main text.

First, the arbitrator's verdict on each disputed dimension equals the pre-arbitration arithmetic mean rather than the outlier reading. On Investigative, two experts voted 2.5 and one voted 4.0; the mean is 3.00 and the arbitrator's verdict is 3.0. On Social, two experts voted 3.5 and one voted 5.0; the mean is 4.00 and the arbitrator's verdict is 4.0. This single-case pattern is consistent with the aggregate finding in Section 3.6 and with the interpretation in Section 4.5. The net effect of arbitration on this case is to endorse the pre-arbitration mean rather than to adopt the outlier or to push the score further toward the scale center.

Second, this case illustrates how arbitration can concentrate on lower-ranked dimensions rather than on the dominant ones. *Director* sits at the Artistic–Enterprising apex (pre-arbitration means A = 6.83, E = 5.83). The three experts agreed on these two dominant RIASEC dimensions as well as on all five Big Five dimensions. Disagreement was confined to two lower-ranked RIASEC dimensions (I, S) that sit at the margin of the *daoyan* taxonomy's scope. The two Holland-code rules record this pattern differently. The proportional-threshold rule retains only dimensions above 0.17 × total (4.216 for this profile). It drops S (4.0) below the baseline and yields **AE**, a two-letter dominance code. The standard top-3 rule, which always returns the three highest-ranked dimensions irrespective of separation, yields **AES**. The gap between the two codes is the boundary signal: S is ranked third but does not clear the equal-weight baseline. Whether arbitration concentrates on lower-ranked dimensions more generally across the 309 arbitrated occupations is a sample-level question, addressed in Section 3.6 of the main text.

Third, the anchor was integrated without being reproduced. The final scores deviate from the anchor in directions that the raters' justifications make explicit. The largest RIASEC deltas are on Investigative (+0.83) and Enterprising (+0.66). Each rater's per-dimension reason in the JSON cites Chinese-taxonomy-specific content. The vocational psychologist's I rationale states "需要深入分析剧本、角色与艺术形式" ("requires deep analysis of script, roles, and artistic form"). The HR specialist's E rationale states "统领剧组、决策选角及营销" ("leads the production team, casting, and marketing"). The largest Big Five delta is on Neuroticism (−0.81). All three raters' N rationales cite ideal-worker emotional stability (e.g., "高压片场需冷静决策与情绪稳定") rather than incumbent stress, mirroring the Case 1 pattern. The profile is anchor-calibrated, not anchor-dictated.

The two per-occupation JSON records are available in the project OSF repository at https://osf.io/gdjb4/ (under occ-psych-agent/output/, organized by Chinese occupation name). Each folder contains seven node-level JSON records (01_Node1_Vector_Retrieval.json through 07_Node7_Finalization.json), the assembled final_profile.json, and the summary.json. The tables in this supplement were produced directly from these files without post-hoc editing; readers who wish to audit the trace at finer granularity (individual justifications, reranker scores, inverse-distance weights, confidence indices) can do so from the JSONs.
