# Supplementary material for: Multi-Agent LLMs for Occupational Profiling: Psychometric Validation on 1636 Chinese Occupations
Source: Behav Sci (Basel). 2026 Jun 26;16(7):1064. doi: 10.3390/bs16071064 (PMC13405925; doi:10.3390/bs16071064)
Supplement: Supplementary file 1 [file behavsci-16-01064-s001.zip › Supplementary_Material_S3.pdf]

Supplementary Material S3: Cluster and Rater-Bias Analyses

This supplement bundles two extended analyses referenced in the main Results, together with two supporting figures and an auxiliary variance-ratio table. S3.1 reports the *k*-means cluster analysis cited in Results §3.4 (Known-group effects). S3.2 reports the rater-model systematic-bias analysis cited in Results §3.6 (Model systematic bias), comprising the per-dimension repeated-measures ANOVA and the Bonferroni-corrected pairwise Hedges' *g*. S3.3 provides the within- to between-occupation variance-ratio table cited in Results §3.2 (MAD analysis). Figure S4 supports the ICC-versus-MAD discussion in Results §3.2; Figure S5 supports the per-rater bias profile cited in Results §3.6.

All data-source references below are relative to the OSF repository root (https://osf.io/gdjb4/), specifically the multi-agent-occupation-profiling/analysis/output/ folder for analysis outputs.

S3.1 Cluster Analysis

S3.1.1 Method

K-means clustering was performed on the 11 *z*-standardized psychological dimensions (six RIASEC and five Big Five) across the $N = 1{,}636$ occupations. The number of clusters was chosen by maximizing the silhouette coefficient over $k = 2$ through $k = 10$. Agreement with the eight-category administrative taxonomy of the 2022 Chinese Occupational Classification was quantified by the adjusted Rand index (ARI).

S3.1.2 Summary statistics

The silhouette coefficient peaked at $k = 5$ (silhouette = .305). The adjusted Rand index between the 5-cluster solution and the 8-category administrative taxonomy was .418.

Three auxiliary figures support the $k = 5$ solution. Figure S1 plots the silhouette coefficient across $k = 2$ through $k = 10$. Figure S2 shows the hierarchical dendrogram of the 1,636 occupations on the 11 *z*-standardized dimensions for visual inspection of cluster structure. Figure S3 shows cluster-level radar profiles for the five-cluster solution.

Figure S1

Silhouette Coefficient across k = 2 to k = 10 for the k-Means Cluster Analysis

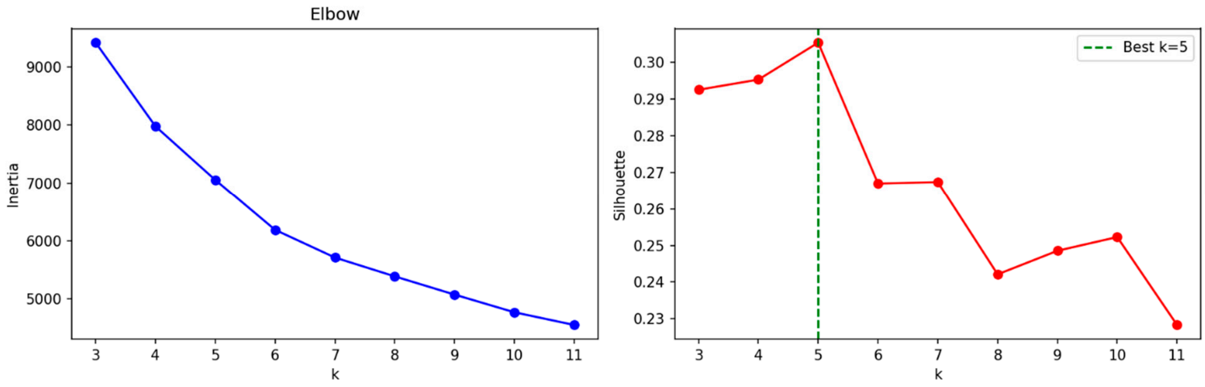

Note. The silhouette coefficient peaks at $k = 5$ (silhouette = .305), the value adopted for the cluster solution reported in Table S3.1.

Figure S2

Hierarchical Dendrogram of the 1,636 Occupations on the 11 z-Standardized Psychological Dimensions

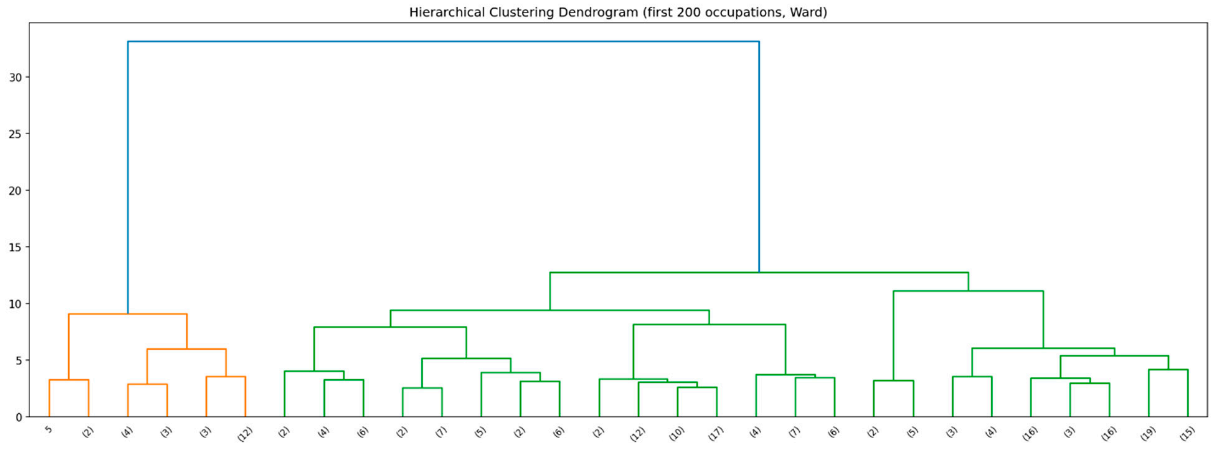

Note. Provided for visual inspection of cluster structure; the *k*-means partition (Table S3.1) is a separate computation.

Figure S3

Cluster-Level Radar Profiles for the Five-Cluster Solution Across the 11 Psychological Dimensions

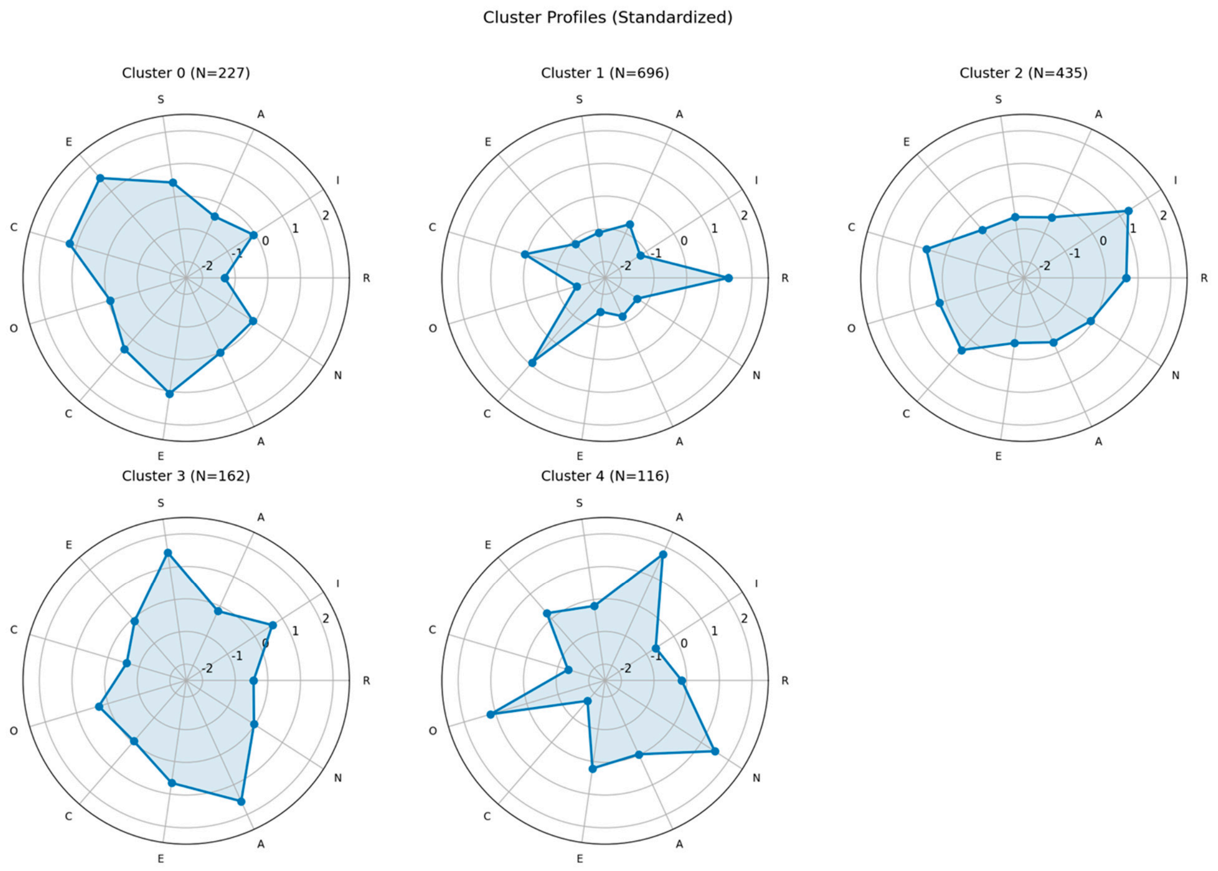

Note. Each panel shows the mean profile of one cluster on the 11 *z*-standardized dimensions. Cluster labels follow the assignments in Table S3.1.

Table S3.1

Cluster Profiles on 11 Psychological Dimensions

Cluster	*n*	RIASEC						Big Five				
		R	I	A	S	E	C	O	C	E	A	N
1: Production / Manufacturing	696	6.74	2.65	1.47	1.56	1.40	4.77	2.71	4.53	2.27	3.12	1.72
2: Technical / Research	435	5.65	5.32	1.90	2.35	2.16	5.19	3.72	4.43	2.75	3.47	1.91
0: Managerial / Service	227	2.31	3.89	1.97	4.11	4.95	5.67	3.56	4.43	3.53	3.61	1.91
3: Social Service	162	3.82	4.62	2.46	5.79	2.78	4.26	3.77	4.34	3.33	4.23	1.92
4: Artistic / Creative	116	4.31	3.25	6.04	3.05	3.20	3.71	4.26	4.03	3.10	3.59	2.15

Note. Rows ordered by cluster size *n*. Cluster labels are interpretive summaries based on the highest-loading dimensions and are not prescribed categories.

S3.1.3 Interpretation

The five-cluster solution separates occupations along the highest-loading dimensions. Three representative occupations per cluster (closest to the cluster centroid in the 11-dimensional standardized space) are listed below.

Cluster 1 (*n* = 696) covers production and manufacturing occupations with extreme Realistic loadings and low Social and Enterprising loadings. Representative examples include maltster (麦芽制麦工), metal heat-treatment worker (金属热处理工), and relay manufacturing technician (继电器制造工).

Cluster 2 (*n* = 435) covers technical and research occupations with jointly high Realistic and Investigative loadings. Representative examples include aircraft manufacturing engineer (飞行器制造工程技术人员), environmental monitoring engineer (环境监测工程技术人员), and instrumentation engineer (仪器仪表工程技术人员).

Cluster 0 (*n* = 227) covers managerial and clerical-service occupations with the highest Conventional and Enterprising loadings. Representative examples include project manager (项目管理工程技术人员), insurance broker (保险经纪人), and labor-dispute arbitrator (劳动人事争议仲裁员).

Cluster 3 (*n* = 162) covers social-service occupations with the highest Social (RIASEC) and Agreeableness (Big Five) loadings. Representative examples include midwife (助产士), Traditional Chinese medicine nurse (中医护士), and dietitian (营养师).

Cluster 4 (*n* = 116) covers artistic and creative occupations with the highest Artistic (RIASEC) and Openness (Big Five) loadings. Representative examples include interior decorator (室内装饰设计师), lighting designer (照明设计师), and furniture designer (家具设计师).

The ARI of .418 indicates that these clusters overlap moderately with, but are not reducible to, the administrative taxonomy.

S3.2 Rater-Model Systematic Bias

S3.2.1 Method

For each of the 11 psychological dimensions, a one-way repeated-measures ANOVA was fit with rater model (DeepSeek, GLM, Kimi) as the within-occupation factor. The reliability sample (N = 1,626 occupations with complete triplets; see Results §3.2) was the analysis set. Effect size for the rater main effect was reported as generalized η^2 (Olejnik & Algina, 2003; Bakeman, 2005), with 90% confidence intervals obtained from 1,000 nonparametric bootstrap resamples of occupations. Post hoc pairwise comparisons used paired t tests with Bonferroni correction across the three contrasts within each dimension. Effect size for each pairwise contrast was Hedges' g.

Table S3.2a

Repeated-Measures ANOVA: Rater Main Effect by Dimension

Dimension	N	F	p	η^2	90% CI	Effect-size descriptor
RIASEC-R	1,626	2.26	.105	.000	[.000, .000]	Negligible
RIASEC-I	1,626	882.86	<.001	.017	[.016, .019]	Small
RIASEC-A	1,626	787.92	<.001	.011	[.010, .013]	Small
RIASEC-S	1,626	1,038.78	<.001	.017	[.015, .018]	Small
RIASEC-E	1,626	346.77	<.001	.005	[.004, .006]	Negligible
RIASEC-C	1,626	44.22	<.001	.002	[.002, .003]	Negligible
BIG FIVE-O	1,626	24.35	<.001	.001	[.001, .002]	Negligible
BIG FIVE-C	1,626	717.76	<.001	.103	[.092, .114]	Medium
BIG FIVE-E	1,626	280.92	<.001	.016	[.013, .019]	Small
BIG FIVE-A	1,626	852.52	<.001	.077	[.069, .085]	Medium
BIG FIVE-N	1,626	1,566.85	<.001	.228	[.209, .248]	Large

Note. df = (2, 3,250) for all F tests. Effect-size bands follow Bakeman (2005).

Table S3.2b

Pairwise Post Hoc Contrasts (Bonferroni-Corrected) by Dimension

Dimension	DeepSeek vs. GLM	DeepSeek vs. Kimi	GLM vs. Kimi
RIASEC-R	−0.010	−.007	.003
RIASEC-I	0.312***	0.196***	−0.121***
RIASEC-A	0.253***	0.134***	−0.126***
RIASEC-S	0.302***	0.219***	−0.085***
RIASEC-E	0.118***	−0.051***	−0.168***
RIASEC-C	0.061***	−0.058***	−0.115***
BIG FIVE-O	0.051***	−0.020	−0.073***
BIG FIVE-C	0.208***	0.751***	0.554***
BIG FIVE-E	0.053***	−0.213***	−0.251***
BIG FIVE-A	0.097***	−0.570***	−0.597***
BIG FIVE-N	−0.143***	−1.317***	−0.996***

Note. Cells report Hedges' *g* with the Hedges correction, computed from paired-samples *t* tests ($df = 1{,}625$). Positive *g* indicates higher scores for the first-named model. *** $p < .001$ (Bonferroni-corrected); cells without asterisks are not significant. 95% CIs for the three largest |*g*| values: Neuroticism vs. DeepSeek [1.25, 1.38]; Conscientiousness vs. DeepSeek [.70, .80]; Agreeableness vs. GLM [.56, .63].

S3.3 Within- to Between-Occupation Variance Ratio

Section 3.2 of the main text reports the within- to between-occupation standard-deviation ratio σ_w/σ_b for three illustrative dimensions (Realistic, Conscientiousness, Neuroticism). Table S3.3 provides the complete ratio for all 11 dimensions, together with the underlying σ_w (mean within-occupation rater *SD* across 1,626 occupations) and σ_b (between-occupation *SD* of dimension means). The ratio diagnoses why low ICC(2,1) can co-occur with low MAD: a small

σ_b shrinks the denominator of ICC, which equals $\sigma_b^2 / (\sigma_b^2 + \sigma_w^2)$, so when between-occupation variance is restricted, ICC is attenuated even when σ_w is itself small.

Table S3.3

Within- to Between-Occupation Standard-Deviation Ratios for the 11 Dimensions

**Dimension**	$\boldsymbol{\sigma_w}$	$\boldsymbol{\sigma_b}$	$\boldsymbol{\sigma_w/\sigma_b}$
RIASEC-R	0.183	1.832	0.100
RIASEC-I	0.348	1.537	0.226
RIASEC-A	0.251	1.395	0.180
RIASEC-S	0.321	1.484	0.216
RIASEC-E	0.244	1.386	0.176
RIASEC-C	0.240	0.772	0.311
BIG FIVE-O	0.210	0.645	0.326
BIG FIVE-C	0.153	0.207	0.739
BIG FIVE-E	0.204	0.560	0.364
BIG FIVE-A	0.245	0.427	0.574
BIG FIVE-N	0.193	0.187	1.025

Note. σ_w = mean within-occupation rater *SD*. σ_b = between-occupation *SD* of the dimension means (as reported in Tables 1 and 2 of the main text). Values < 0.30 indicate between-occupation variation dominates; values ≥ 1 indicate within-occupation rater dispersion equals or exceeds between-occupation variation.

Figure S4

ICC(2,1) versus Mean Absolute Deviation Across the 11 Psychological Dimensions for the 1,626 Occupations with Complete Triplets

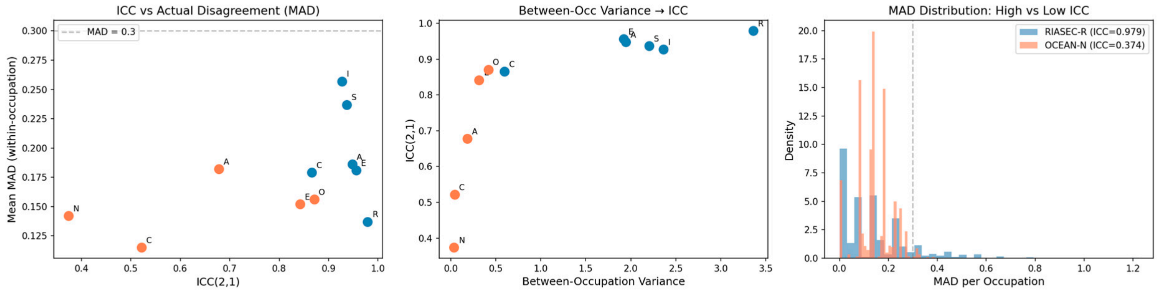

Note. The low Big Five ICCs reflect restricted between-occupation variance rather than high rater disagreement.

Figure S5

Per-Rater Marginal-Mean Profile for DeepSeek, GLM, and Kimi Across the Six RIASEC and Five Big Five Dimensions

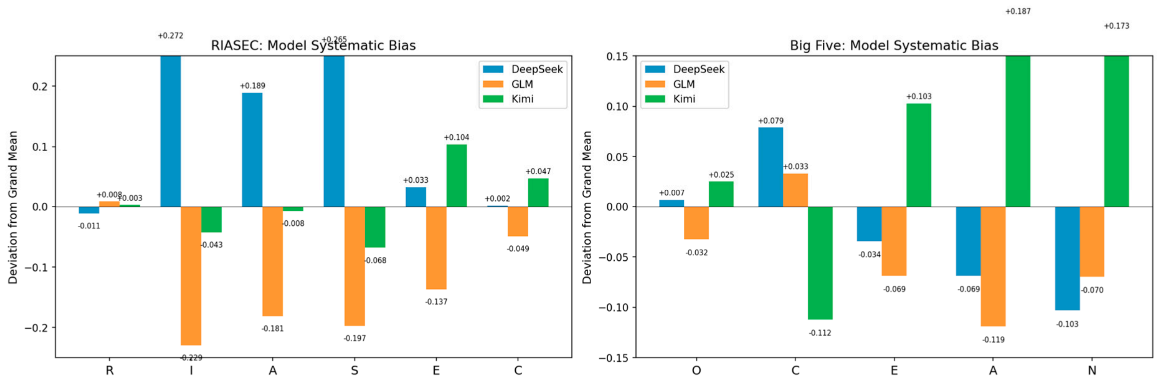

Note. The grand mean is overlaid for comparison. Kimi's upward shifts on Neuroticism (+0.17 vs. grand mean) and Agreeableness (+0.19) and downward shift on Conscientiousness (−0.11) stand out; all RIASEC departures fall below 0.3 on the 1–7 scale. Shifts are raw marginal-mean differences (1–7 RIASEC, 1–5 Big Five), not standardized effect sizes; standardized contrasts are in Table S3.2b.

References (Supplementary)

Bakeman, R. (2005). Recommended effect size statistics for repeated measures designs. *Behavior Research Methods*, *37*(3), 379–384. https://doi.org/10.3758/BF03192707

Olejnik, S., & Algina, J. (2003). Generalized eta and omega squared statistics: Measures of effect size for some common research designs. *Psychological Methods*, *8*(4), 434–447. https://doi.org/10.1037/1082-989X.8.4.434
