# Supplementary material for: Multi-Agent LLMs for Occupational Profiling: Psychometric Validation on 1636 Chinese Occupations
Source: Behav Sci (Basel). 2026 Jun 26;16(7):1064. doi: 10.3390/bs16071064 (PMC13405925; doi:10.3390/bs16071064)
Supplement: Supplementary file 1 [file behavsci-16-01064-s001.zip › Supplementary_Material_S4.pdf]

Supplementary Material S4: Supporting Data for Big Five Rescaling, MTMM, and Cross-Cultural Comparison

This supplement reports the inputs and full output tables for three analyses summarized in the main text. S4.1 documents the rescaling from Anni et al.'s (2025) Estonian *T* metric to the Chinese BFI-2 1–5 metric used as the Big Five anchor (cited in Method §2.1 and Results §3.5). S4.2 reproduces the full 18 × 18 multitrait–multimethod correlation matrix and verifies the four Campbell-and-Fiske criteria at the cell level (cited in Results §3.3 and Discussion §4.1). S4.3 reports the cross-cultural China-vs.-U.S. comparison for all six RIASEC dimensions, complementing the three flagged dimensions in Results §3.6 and Discussion §4.3.

All file references below are relative to the OSF repository root (https://osf.io/gdjb4/). Anchor and data files live under multi-agent-occupation-profiling/occ-psych-agent/data/; analysis outputs live under multi-agent-occupation-profiling/analysis/output/.

S4.1 BFI-2 Chinese Rescaling

S4.1.1 Rationale

Method §2.1 states that Anni et al.'s (2025) smoothed residualized Big Five scores, originally on the Estonian *T* metric ($M = 50$, $SD = 10$), were rescaled onto the Chinese working-adult BFI-2 1–5 metric using Zhang et al.'s (2022) employee-sample norms ($n = 486$). Because Pearson correlations are invariant to linear rescaling, the specific values of M_{China} and SD_{China} do not directly determine the convergent-validity *r* reported in Results §3.5. They do, however, determine two other quantities that the main paper relies on: the absolute anchor values delivered to the scoring agents during rating (Method §2.3), and the Chinese-scale mean-difference analysis in Results §3.5. The Conscientiousness offset of +0.69 and the Neuroticism offset of −0.79 reported there are both expressed on the 1–5 metric produced by this rescaling.

Table S4.1

Zhang et al. (2022) Chinese Employee-Sample Domain Norms for the BFI-2 (n = 486)

**BFI-2 domain**	M_{China}	SD_{China}	α
Extraversion	3.24	0.60	.85
Agreeableness	3.81	0.49	.81
Conscientiousness	3.68	0.57	.86
Neuroticism	2.72	0.61	.86
Openness	3.52	0.57	.83

Note. The five M_{China} and SD_{China} values are the parameters applied to every Anni et al. source score during rescaling. The "Neuroticism" label follows Zhang et al.; the BFI-2 official label is "Negative Emotionality." Cronbach's α is reported for documentation only and is not used in the rescaling. Source: Zhang et al. (2022), Table 1, column "CN employee."

S4.1.2 Rescaling formula

For every Anni et al. occupation × dimension cell, the rescaling proceeds in two steps. The rescaling proceeds in two steps: $Z = (T - 50) / 10$, then $S = Z \times SD_{\text{China}} + M_{\text{China}}$. Here T is the source T-score, SD_{China} and M_{China} are the domain-specific values in Table S4.1, and S is the rescaled Chinese-metric score.

S4.2 Full 18 × 18 MTMM Correlation Matrix

Table S4.2

18 × 18 Multitrait–Multimethod Correlation Matrix (3 Raters × 6 RIASEC Traits, N = 1,626 Occupations With Complete Triplets)

	DS_R	DS_I	DS_A	DS_S	DS_E	DS_C	GLM_R	GLM_I	GLM_A	GLM_S	GLM_E	GLM_C	K_R	K_I	K_A	K_S	K_E	K_C
DS_R	1.00	−.35	−.34	−.74	−.80	−.26	**.98**	−.26	−.30	−.73	−.80	−.20	**.98**	−.27	−.32	−.72	−.81	−.23
DS_I	−.35	1.00	.08	.28	.27	.22	−.36	**.95**	.04	.26	.23	.25	−.36	**.95**	.04	.32	.26	.23
DS_A	−.34	.08	1.00	.29	.30	−.35	−.38	.04	**.96**	.29	.29	−.47	−.38	.01	**.96**	.28	.33	−.41
DS_S	−.74	.28	.29	1.00	.65	−.01	−.73	.21	.24	**.96**	.67	−.09	−.74	.24	.27	**.96**	.65	−.07
DS_E	−.80	.27	.30	.65	1.00	.17	−.80	.16	.25	.60	**.97**	.12	−.80	.19	.28	.59	**.96**	.17
DS_C	−.26	.22	−.35	−.01	.17	1.00	−.23	.13	−.39	−.04	.16	**.88**	−.24	.16	−.38	−.03	.19	**.85**
GLM_R	**.98**	−.36	−.38	−.73	−.80	−.23	1.00	−.27	−.34	−.73	−.80	−.17	**.98**	−.28	−.36	−.71	−.81	−.20
GLM_I	−.26	**.95**	.04	.21	.16	.13	−.27	1.00	.02	.21	.13	.20	−.27	**.96**	.01	.27	.15	.18
GLM_A	−.30	.04	**.96**	.24	.25	−.39	−.34	.02	1.00	.24	.24	−.51	−.34	−.02	**.97**	.23	.28	−.45
GLM_S	−.73	.26	.29	**.96**	.60	−.04	−.73	.21	.24	1.00	.64	−.12	−.73	.23	.27	**.97**	.61	−.11
GLM_E	−.80	.23	.29	.67	**.97**	.16	−.80	.13	.24	.64	1.00	.12	−.80	.15	.27	.62	**.96**	.16
GLM_C	−.20	.25	−.47	−.09	.12	**.88**	−.17	.20	−.51	−.12	.12	1.00	−.18	.23	−.49	−.10	.14	**.89**
K_R	**.98**	−.36	−.38	−.74	−.80	−.24	**.98**	−.27	−.34	−.73	−.80	−.18	1.00	−.28	−.37	−.72	−.81	−.20
K_I	−.27	**.95**	.01	.24	.19	.16	−.28	**.96**	−.02	.23	.15	.23	−.28	1.00	−.02	.30	.18	.22
K_A	−.32	.04	**.96**	.27	.28	−.38	−.36	.01	**.97**	.27	.27	−.49	−.37	−.02	1.00	.27	.31	−.44
K_S	−.72	.32	.28	**.96**	.59	−.03	−.71	.27	.23	**.97**	.62	−.10	−.72	.30	.27	1.00	.61	−.08
K_E	−.81	.26	.33	.65	**.96**	.19	−.81	.15	.28	.61	**.96**	.14	−.81	.18	.31	.61	1.00	.18
K_C	−.23	.23	−.41	−.07	.17	**.85**	−.20	.18	−.45	−.11	.14	**.89**	−.20	.22	−.44	−.08	.18	1.00

Note. DS = DeepSeek, GLM = GLM, K = Kimi. Bold cells identify the monotrait-heteromethod block (same trait across different raters, n = 18); these are the convergent-validity coefficients. The three off-block 6 × 6 inter-method submatrices contain heterotrait-heteromethod values (different trait *and* different rater, n = 90). The three diagonal 6 × 6 within-method submatrices contain heterotrait-monomethod values (different trait within the same rater, n = 45). Cell values are rounded to two decimals.

Criterion 1, convergent validity. All 18 monotrait-heteromethod correlations exceed zero, with values from .85 (DS_C × K_C) to .98 (DS_R × GLM_R = DS_R × K_R = GLM_R × K_R). The median is .96 and the minimum is .85 (Conventional, Kimi vs. DeepSeek). Satisfied.

Criterion 2, discriminant validity I (monotrait > heterotrait-heteromethod). Each monotrait-heteromethod coefficient should exceed the heterotrait-heteromethod coefficients sharing its row or column. With monotrait values in [.85, .98] and the 90 heterotrait-heteromethod values in [−.81, .67] (mean = −.060), every monotrait cell exceeds every heterotrait-heteromethod cell in its row or column. Block-mean contrast Δ = 1.01, Cohen's d = 3.64. Satisfied.

Criterion 3, discriminant validity II (monotrait > heterotrait-monomethod). Each monotrait-heteromethod cell should exceed in absolute value the heterotrait-monomethod coefficients involving its trait within each contributing method. Defining margin as monotrait minus the larger of the two method-specific maxima of |heterotrait-monomethod| on the same trait, the 18 margins range from .15 (Extraversion, where within-method |E × R| ≈ .80–.81 dominates) to .41 (Conventional, whose within-method heterotrait correlations are themselves modest, max |C × A| ≈ .51 in GLM). All 18 cells pass; the smallest margin is .15. Satisfied.

Criterion 4, same pattern of trait intercorrelations across methods. Each method's 6 × 6 heterotrait-monomethod submatrix yields a 15-element vector of unique trait intercorrelations. Pairwise correlations between these vectors are nearly identical across the three methods (Fisher r-to-z 95% CIs in Table S4.3).

Table S4.3

Pairwise Correlations Between the Three Methods' 15-Element Trait-Intercorrelation Vectors

Pair	*r*	95% CI
DeepSeek vs. GLM	.986	[.957, .995]
DeepSeek vs. Kimi	.992	[.975, .997]
GLM vs. Kimi	.995	[.985, .998]

S4.3 Cross-Cultural Comparison Across All Six RIASEC Dimensions

Table S4.4

*China (LLM) vs. U.S. (O*NET) RIASEC Comparison Across the Six Dimensions on the 1,539 Occupations With Top-1 O*NET Match Score ≥ .85*

Dimension	China *M* (LLM)	U.S. *M* (O*NET)	Mean difference	Cohen's *d*	*t*	*p*
Realistic	5.42	5.34	+0.07	0.14	5.36	< .001
Investigative	3.75	3.33	+0.43	0.60	23.59	< .001
Artistic	2.06	1.93	+0.14	0.25	9.61	< .001
Social	2.61	2.16	+0.44	0.84	33.04	< .001
Enterprising	2.34	2.37	−0.03	−0.06	−2.24	.025
Conventional	4.88	4.46	+0.42	0.93	36.46	< .001

Note. Means are on the 1–7 RIASEC scale. Positive mean differences indicate higher Chinese (LLM) scores than matched U.S. O*NET scores. Paired-samples *t* tests, *df* = 1,538.

The pattern separates into two halves. On Social, Conventional, and Investigative, the Chinese LLM scores lie about 0.42 to 0.44 above the matched O*NET anchor, with medium-to-large effect sizes ($|f| \geq 0.60$). On Realistic, Artistic, and Enterprising, the LLM scores differ by less than 0.15 of a scale point, with small (Artistic $d = 0.25$) or trivial (Realistic $d = 0.14$; Enterprising $d = -0.06$) effects. With $N = 1,539$ matched occupations, the paired t tests reach significance even on the trivial-effect dimensions, so the appropriate reading is effect-size magnitude rather than statistical significance.

This bimodal pattern is consistent with the Discussion §4.3 reading that the agents adjust the anchor when Chinese occupational content warrants and otherwise leave it largely intact. The trivial effects on Realistic, Artistic, and Enterprising show that the agents do retain the anchor when content does not warrant departure, which is stronger evidence against a uniform-upward-drift interpretation than the three large-d dimensions alone. The pattern does not uniquely identify Chinese occupational content as the driver; alternatives discussed in Discussion §4.3 remain compatible.

References (Supplementary)

Anni, K., Vainik, U., & Mõttus, R. (2025). Personality profiles of 263 occupations. *Journal of Applied Psychology*, *110*(4), 481–511. https://doi.org/10.1037/apl0001249

Campbell, D. T., & Fiske, D. W. (1959). Convergent and discriminant validation by the multitrait-multimethod matrix. *Psychological Bulletin*, *56*(2), 81–105. https://doi.org/10.1037/h0046016

Zhang, B., Li, Y. M., Li, J., Luo, J., Ye, Y., Yin, L., Chen, Z., Soto, C. J., & John, O. P. (2022). The Big Five Inventory-2 in China: A comprehensive psychometric evaluation in four diverse samples. *Assessment*, *29*(6), 1262–1284. https://doi.org/10.1177/10731911211008245
